# Supplementary material for: Inhibition of CSF-1R and IL-6R prevents conversion of cDC2s into immune incompetent tumor-induced DC3s boosting DC-driven therapy potential
Source: Cell Rep Med. 2024 Jan 18;5(2):101386. doi: 10.1016/j.xcrm.2023.101386 (PMC10897516; doi:10.1016/j.xcrm.2023.101386)
Supplement: Document S1. Figures S1–S6 and Tables S1–S4 and S6 [file mmc1.pdf]

Cell Reports Medicine, Volume *5*

Supplemental information

Inhibition of CSF-1R and IL-6R prevents conversion of cDC2s into immune incompetent tumor-induced DC3s boosting DC-driven therapy potential

Anouk M.D. Becker, Annika H. Decker, Georgina Flórez-Grau, Ghaith Bakdash, Rutger J. Röring, Suzan Stelloo, Michiel Vermeulen, Berber Piet, Erik H.J.G. Aarntzen, Martijn Verdoes, and I. Jolanda M. de Vries

Supplemental information

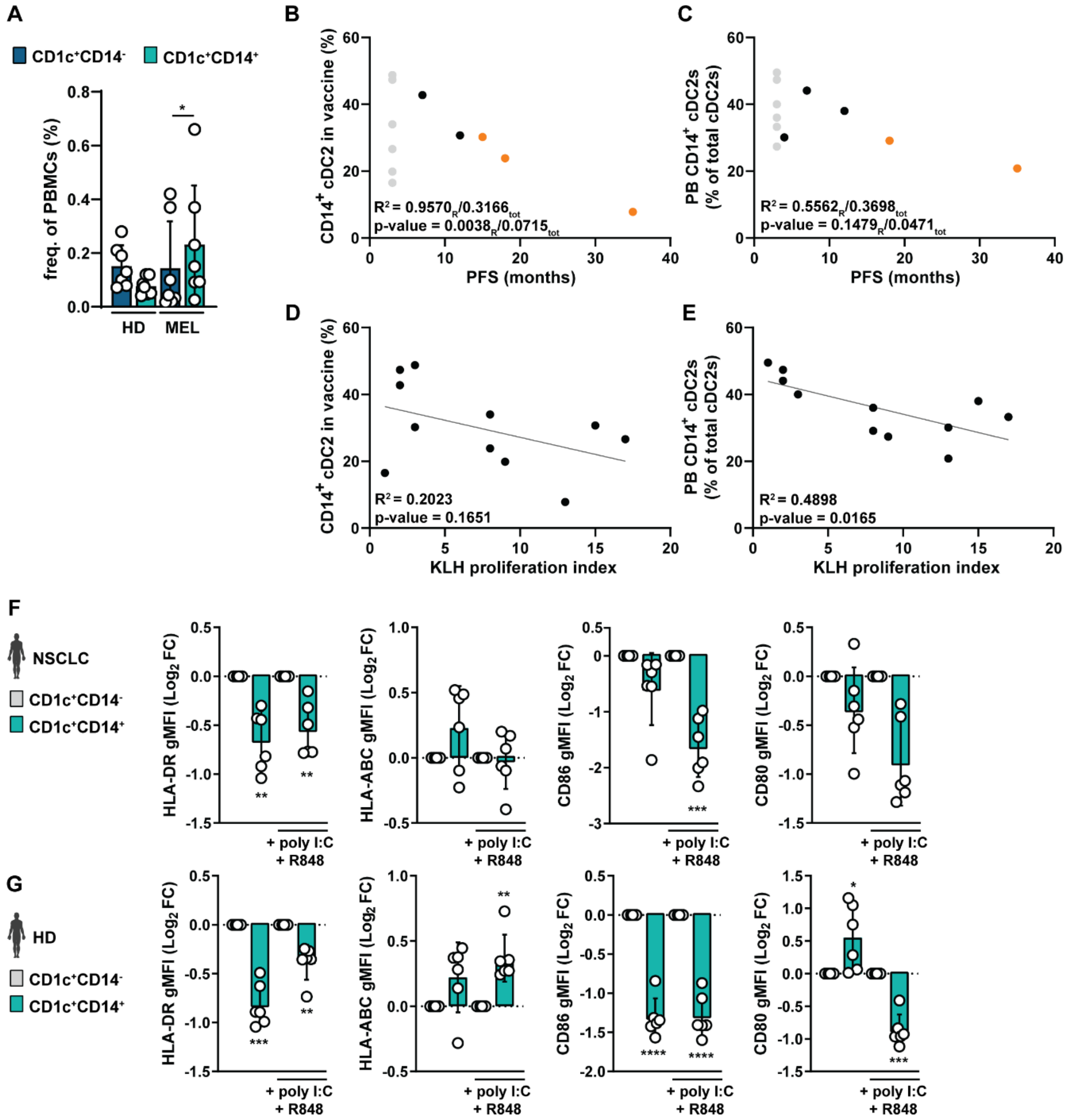

Figure S1. CD1c$^+$CD14$^+$ DCs affect DC vaccination efficacy and KLH-specific T cell proliferation in melanoma patients, and have lower co-stimulatory markers compared to CD1c$^+$CD14$^-$ cells in lung cancer patients and HDs. Related to Figure 1.

A Frequencies of CD1c$^+$CD14$^-$ and CD1c$^+$CD14$^+$ within PBMCs of healthy donors (HD) and melanoma patients (MEL). Each symbol shows a biological replicate with n=7 per group (mean±SD), two-way ANOVA with Sidak's multiple comparison test. **B** The fraction of CD1c$^+$CD14$^+$ in CD1c$^+$ DC-based vaccinations measured by flow cytometry for melanoma patients undergoing CD1c$^+$ DC-based vaccinations, correlated with the months of progression-free survival (PFS). **C** Correlation of PFS with the fraction of CD1c$^+$CD14$^+$ cells of total CD1c$^+$ (cDC2s) cells in peripheral blood (PB) of the same melanoma patients. Orange depicts patients with functional tumor antigen-specific T cells detected in peripheral blood or skin-infiltrating lymphocytes, grey indicates non-responder patients (all showing progressive disease). Correlation was statistically tested with Pearson's correlation coefficient and two-tailed P value, reported for responding patients($_R$)/all patients ($_{tot}$). Pearson's correlation coefficient of the anti-keyhole limpet hemocyanin (KLH) specific T cell proliferation from melanoma patients with **D)** the percentage of CD1c$^+$CD14$^+$ in CD1c$^+$ DC-based vaccinations and in **E)** the correlation with the fraction of CD1c$^+$CD14$^+$ cells of total CD1c$^+$ (cDC2s) cells in peripheral blood (PB) of melanoma patients. **B-D**

n=11 biological replicates (patients). PBMCs from **F)** NSCLC patients and **G)** healthy donors were depleted for CD3, CD19, CD56, using MACS beads followed by positive selection of $CD1c^+$ cells using the CD1c (BDCA1) DC isolation kit (Miltenyi). $CD1c^+$ cells were FACS sorted into $CD1c^+CD14^-$ and $CD1c^+CD14^+$ cells, cultured overnight with or without 20 µg/mL poly I:C and 4 µg/mL R848 and subsequently analyzed by flowcytometry for the surface expression of HLA-DR, HLA-ABC, CD86, CD80. Graphs show Log_2 FC of $CD1c^+CD14^+$ compared to $CD1c^+CD14^-$ cells, for immature and mature cDC2s. Each symbol represents an individual donor (mean±SD), with n=6 biological replicates for NSCLC and HD. Asterisks depict significance compared to $CD1c^+CD14^-$ cells in the same condition (two-tailed paired T-test), *p<0.05, **p<0.01, ***p<0.001, ****p<0.0001.

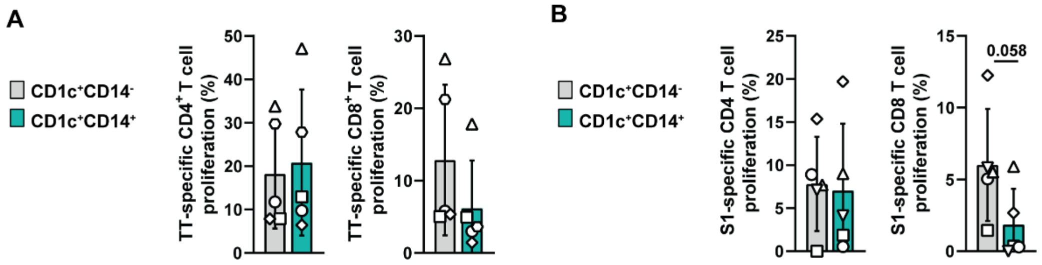

Figure S2. Antigen processing and presentation of cDC2 subsets in antigen-specific recall responses. Related to Figure 2.

$CD1c^+CD14^-$ and $CD1c^+CD14^+$ cells were isolated by FACS from healthy donors, rested overnight, and subsequently cultured with or without 1 µg/ml Tetanus Toxoid (TT, Sigma Aldrich) **(A)** or SARS-CoV-2 S Protein S1 (S1, Biolegend, 792904) **(B)** for 24 h. After 24 h, DCs were washed twice and co-cultured with CFSE-labelled autologous human pan T-cells (DC:T-cell ratio of 1:25) for 6 days after which T-cell proliferation was analyzed by flow cytometry. Antigen-specific T-cell proliferation was calculated by subtracting the percentage of proliferating T-cells measured in the conditions without antigen from the TT/S1 condition. Only donors showing an antigen-specific recall response were included in the analysis, each donor is represented by a different symbol with n=5 biological replicates each representing the average of technical duplicates (mean±SD, paired T-test).

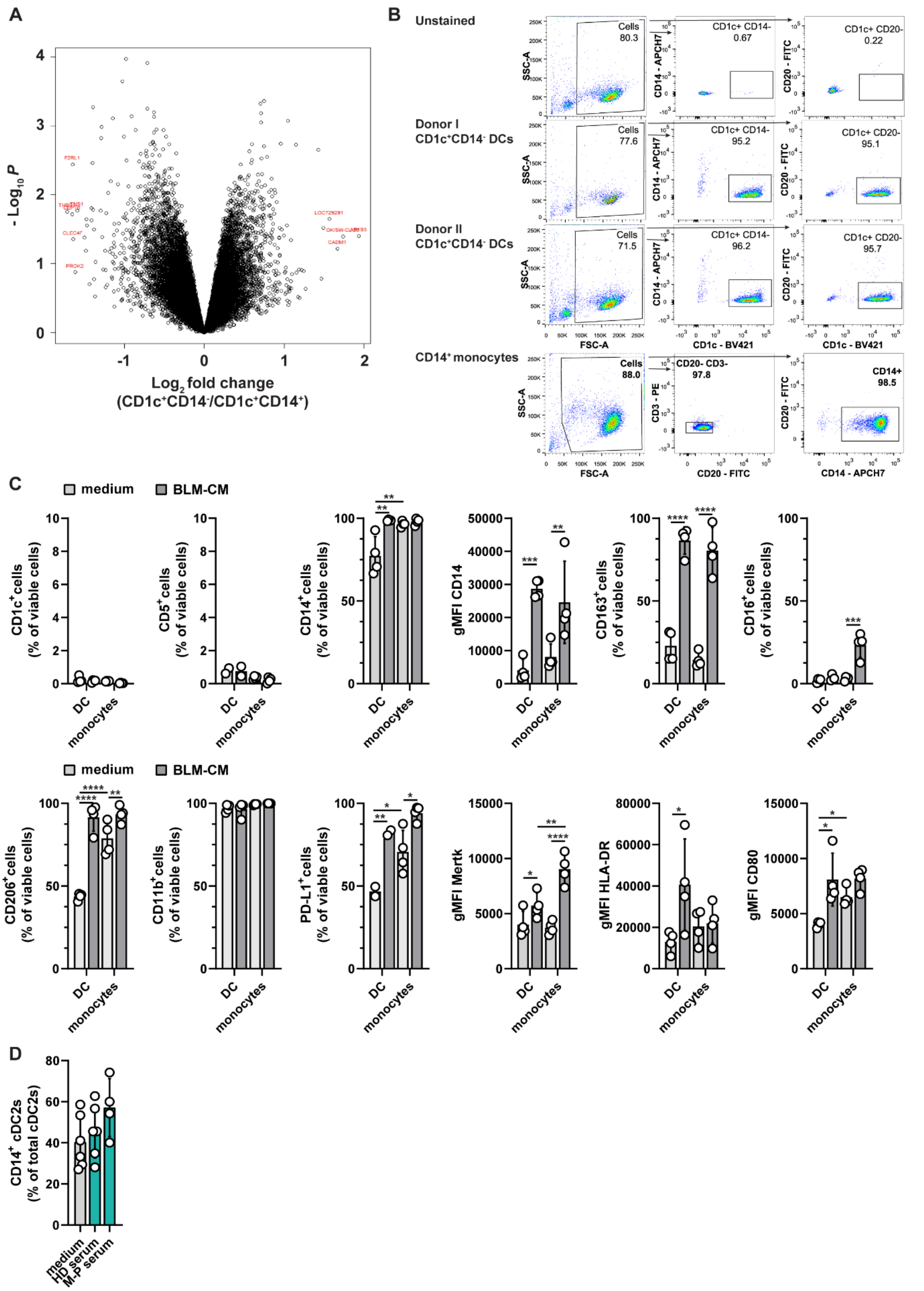

Figure S3. Tumor-induced CD1c^{+}CD14^{+} cells derived from cDC2s phenotypically resemble macrophages after longer culture periods. Related to Figure 3.

A Volcano plot displaying expressed genes between CD1c^{+}CD14^{-} and CD1c^{+}CD14^{+} cells as analyzed by RNA Affymetrix Array Eurofins using n=4 biological replicates. Genes with an absolute FC > 1.5 are labeled, y-axis

depict non-adjusted P value, no genes were differentially expressed after correction for multiple testing. **B** Representative dot plots of purity analysis of $CD1c^{+}CD14^{-}$ cells and $CD14^{+}$ monocytes freshly isolated from human buffy coats, all stained with primary directly labelled antibodies: CD1c-BV421, CD20-FITC, CD3-PE and CD14-APC-H7 and assessed by flow cytometry. After MACS depletion of $CD19^{+}$ and $CD14^{+}$ cells, $CD1c^{+}$ cells were isolation by positive selection using the CD1c (BDCA1) DC isolation kit (Miltenyi). $CD14^{+}$ monocytes were directly isolated from PBMCs by positive selection using MACS CD14 beads (Miltenyi). **C** $CD14^{+}$ monocytes and $CD1c^{+}CD14^{-}$- DCs were isolated from HDs, cultured for seven days in X-VIVO (2% HS) with or without 50% BLM-conditioned medium (BLM-CM), and analyzed by flow cytometry (See also Table S4). Each symbol represents an individual donor (mean±SD, 2-way ANOVA with Sidak's multiple comparisons test for DCs versus monocytes and medium versus BLM-CM), with n=4 biological replicates. **D,** Frequencies of $CD1c^{+}CD14^{+}$ cells after a three-day culture of $CD14^{-}$ cDC2s from healthy donors (HDs) with HD serum or melanoma patients (M-P) serum. Each symbol represents an individual serum donor or DC donor for the medium condition, with n=6 biological replicates for medium, n=6 for HD serum and n=4 for serum from M-P, each tested on a minimum of n=2 DC-donors.

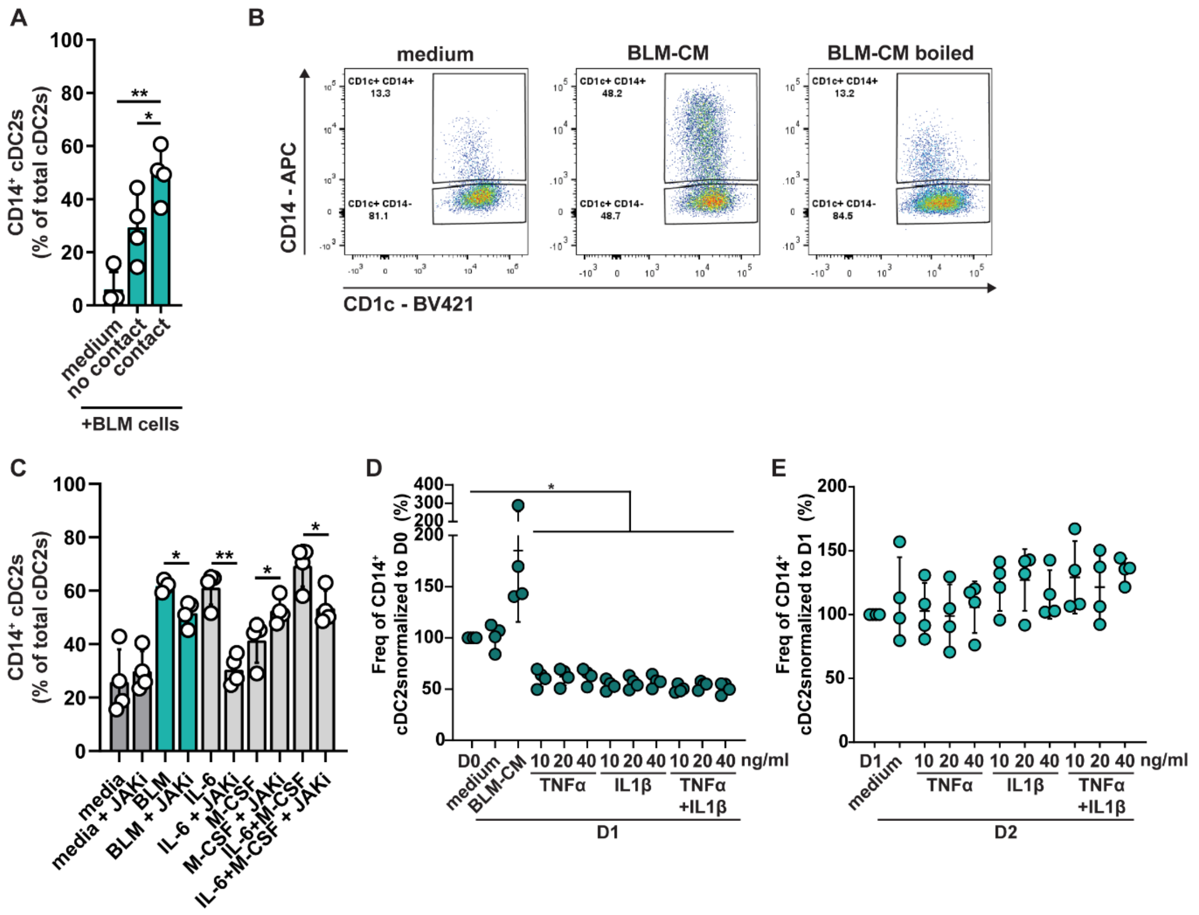

Figure S4. CD14⁺ cDC2s are irreversibly induced by soluble tumor-associated proteins. Related to Figure 4 and 5.

A Frequencies of CD14⁺ cDC2s after culturing CD14⁻ cDC2s from healthy donors in a transwell plate with or without direct cell-cell contact with BLM cells, n=4 biological replicates depicted by each symbol. **B** CD14⁻ cDC2s from healthy donors were cultured in the presence of 40% BLM-conditioned medium (CM) either untreated or heated at 95 °C for 10 minutes. After three days, cultured cells were analyzed by flow cytometry for CD14 and CD1c expression. Shown are representative dot-plots of n=3 biological replicates. **C** Induction of CD14⁺ cDC2s in the absence or presence of 1 µM Janus kinase inhibitor (JAKi) Tofacitinib after a two-day culture period with 50% BLM-CM, 25 ng/mL IL-6 and/or 25 ng/mL M-CSF. Each symbol represents a biological replicate (n=4), mean±SD, one-way RM ANOVA with Dunnett's multiple comparisons test between untreated and JAKi treated for each condition. **D+E** Phenotypic plasticity of CD14⁺cDC2s analyzed for CD14⁺ cDC2s from healthy donors **(D)** and for BLM-CM induced CD14⁺ cDC2s **(E)**. Graphs show frequencies of CD14⁺cDC2s normalized to D0 **(D)** and normalized to D1 after inducing CD14⁺ cDC2s with BLM-CM for 24 hours **(E),** both with the indicated concentration range of TNFα and/or IL1β. Each symbol represents an individual donor, with n=4 biological replicates for each experiment (mean±SD). Asterisks depict significance compared to D0 **(D)** or D1 **(E)** (RM one-way ANOVA, Dunnett's multiple comparisons test). *p<0.05, **p<0.01, ***p<0.001

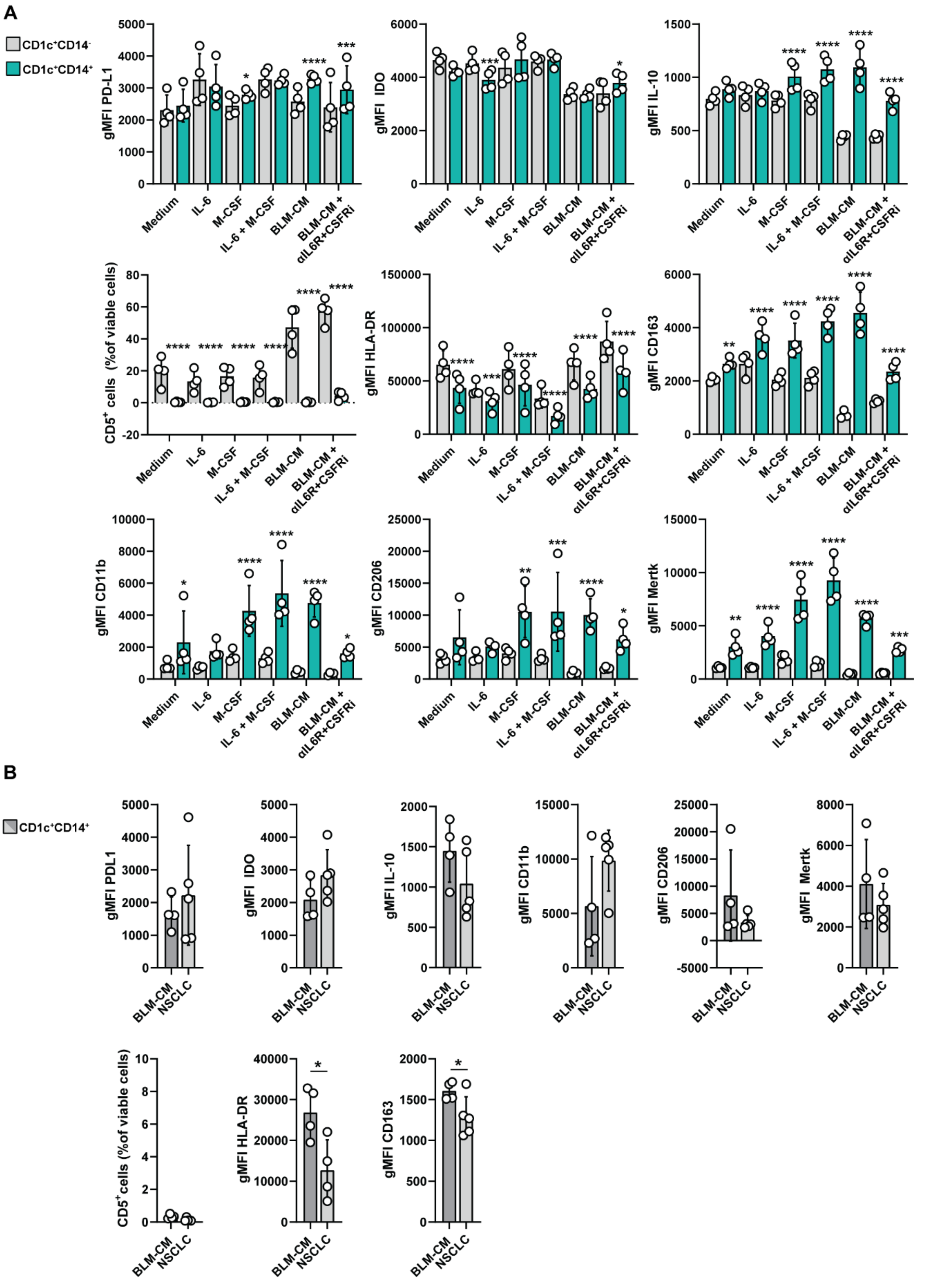

Figure S5. ***In vitro* tumor induced CD14⁺ cDC2s phenotypically resemble CD14⁺ cDC2s from lung cancer patients. Related to Figure 6.**

A $CD14^-$ cDC2s isolated from HDs were cultured for three days in depicted conditions and expression of phenotypic hallmarks was assessed by flow cytometry and compared between induced $CD14^+$ cDC2s and the remaining $CD14^-$ cDC2s in culture. Each symbol represents an individual donor (mean ± SD) with n=4 biological replicates, asterisks depict significant results compared to $CD14^-$ cDC2s (2-way RM ANOVA, Sidak's multiple

comparisons test). **B** Phenotype comparison between BLM-conditioned medium (CM) induced $CD14^+$ cDC2s from HDs and $CD14^+$ cDC2s isolated from NSCLC patients, both cultured for three days (unpaired t-test, HD n=4, NSCLC n=5 biological replicates, mean ± SD). *p<0.05, **p<0.01, ***p<0.001, ****p<0.0001

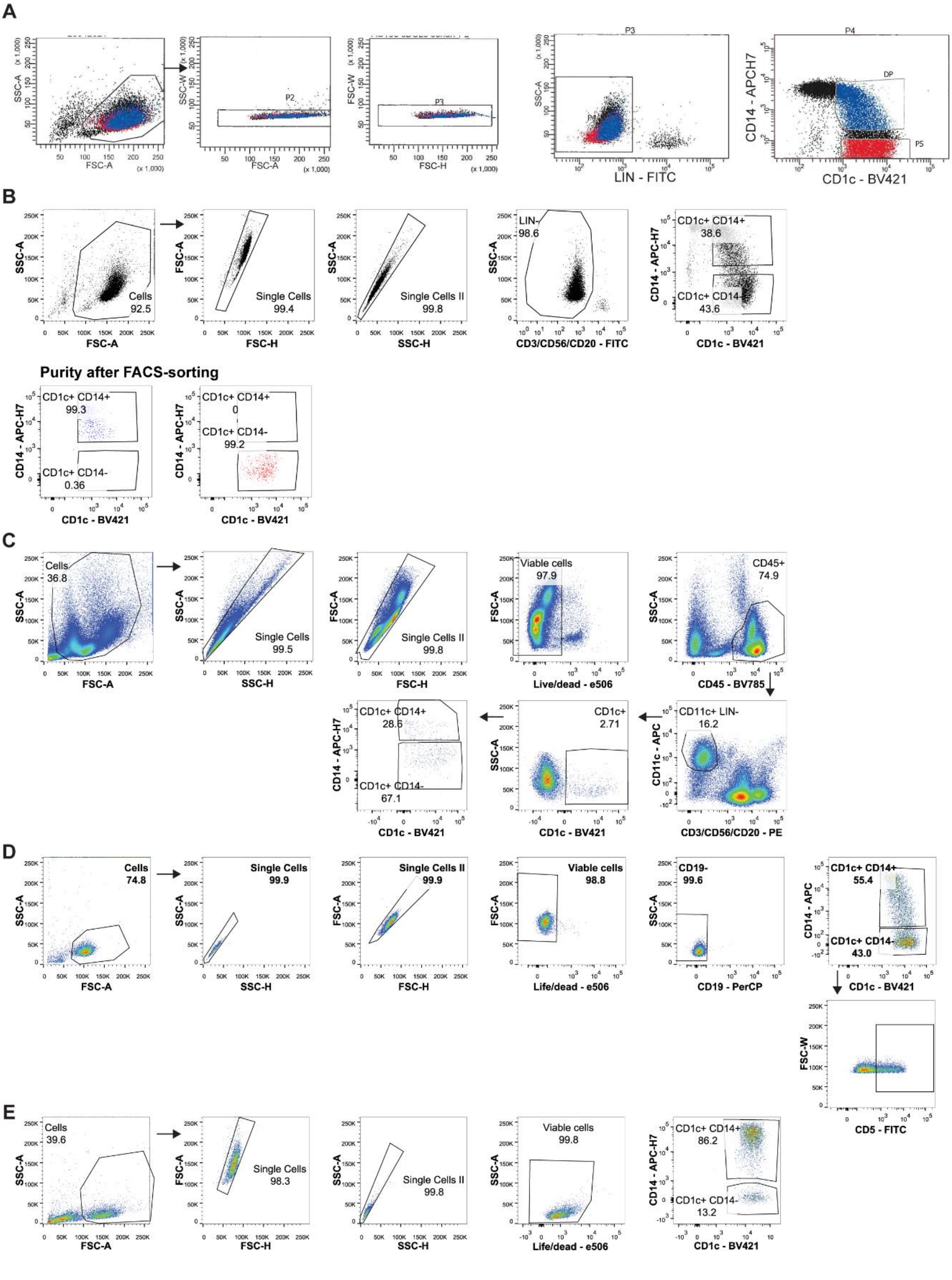

Figure S6. Gating strategies. Related to STAR Methods, Figure 1-6, Table S4.

Manual gating strategy to define and sort CD1c^{+}CD14^{-} and CD1c^{+}CD14^{+} cells after lineage depletion performed on BD FACS Aria **(A)** or Melody **(B)**, with two representative purity plots. Sorted cells were used for bulk RNA microarray analysis, T-cell assays, and cytokine profiling. **C** Gating strategy to define CD1c^{+}CD14^{-} and CD1c^{+}CD14^{+} cells within peripheral blood mononuclear cells. Representative examples of the gating strategy for flow cytometry analysis of isolated CD1c^{+} cells is shown in **D)** for samples immediately stained after isolation (some panels include CD14-APC-H7 and CD20-FITC instead of CD14-APC and CD19-PerCP, respectively, see table S4) and in **E)** for CD1c^{+} cells after a culture period. See also table S4.

Table S1. Patient information melanoma patients DC-vaccination trial. Related to STAR Methods, Fig. S1A-E, 1A.

ID	disease (stage)	gender	year of birth
**Melanoma patients DC-vaccination trial (Fig. S1B-E)**			
VI-B-01	melanoma (M1a)	M	1958
VI-B-02	melanoma (M1c)	M	1945
VI-B-03	melanoma (M1a)	F	1968
VI-B-04	melanoma (M1c)	M	1985
VI-B-05	melanoma (M1c)	F	1974
VI-B-06	melanoma (M1c)	M	1964
VI-B-08	melanoma (M1c)	F	1962
VI-B-09	melanoma (M1a)	M	1947
VI-B-10	melanoma (M1c)	F	1950
VI-B-11	melanoma (M1c)	M	1968
VI-B-12	melanoma (M1b)	M	1970
VI-B-13	melanoma (M1b)	M	1970
VI-B-14	melanoma (IIIcb)	M	1943
**Melanoma patients PBMC assessment (Fig. 1A, Fig. S1A)**			
IPI02	melanoma	F	1942
IPI08	melanoma	F	1961
IPI09	melanoma	F	1946
IPI11	melanoma	M	1958
IPI12	melanoma	M	1952
IPI13	melanoma	F	1963
IPI14	melanoma	F	1945

Table S2. Patient information non-small cell lung cancer (NSCLC) patients. Related to STAR Methods, Fig. 1, 2A-B, 3A-B

ID	disease	gender	year of birth
DON240	NSCLC	F	1968
DON395	NSCLC	F	1969
DON014	NSCLC	F	1948
DON092	NSCLC	M	1947
DON609	NSCLC	F	1969
DON986	NSCLC	M	1964
DON104	NSCLC	F	1962
DON469	NSCLC	F	1949
DON344	NSCLC	M	1952
DON622	NSCLC	F	1963
DON430	NSCLC	M	1949
DON570	NSCLC	F	1957
DON166	NSCLC	F	1948
DON159	NSCLC	M	1948
DON109	NSCLC	M	1944
DON590	NSCLC	M	1957

Table S3. Absolute cytokine amounts produced by CD1c⁺CD14⁻ and CD1c⁺CD14⁺ cells as measured by LEGENDPlex. Related to figure 2A-B. Shown are average results of HD (n=5) and non-small cell lung cancer (NSCLC) patients (n=6). IFNα and IFNγ levels were below detection limits. DC2: $CD1c^{+}CD14^{-}$, DC3: $CD1c^{+}CD14^{+}$

	**IL-6**	**IL-1β**	**TNFa**	**MCP-1**	**IL-8**	**IL-10**	**IL12-p70**	**IL-17**	**IL-18**	**IL-23**	**IL-33**
**HD - DC2**	21.33	3.28	3.09	3.45	5710	2.25	1.30	0.16	10.18	6.16	5.90
**HD - DC3**	2395	380.3	235.10	83.46	12000	104.4	36.64	0.25	27.83	144	7.84
**NSCLC - DC2**	54.93	1.68	4.43	5.15	3287	1.92	1.03	0.13	6.55	5.76	5.00
**NSCLC - DC3**	468.5	62.55	35.73	144.4	4900	14.43	1.38	0.16	10.93	10.5	6.07
**HD mature DC2**	7687	1117	5428	16.24	12000	478.1	758.6	0.39	38.83	1658	17.76
**HD mature DC3**	7766	2880	6938	49.95	12000	392.2	194.1	0.36	51.50	3068	24.94
**NSCLC mature DC2**	4020	541.8	2966	237.10	12000	394.2	467.9	0.21	26.10	837.4	15.59
**NSCLC mature DC3**	5045	1942	4217	1189	12000	379.6	72.3	0.29	44.56	1312	17.38

[a]blue and red indicate lower and upper detection limit, respectively

Table S4. FACS panels used in the study. See also Figure S6.

	Laser		Violet (405nm)				Blue (488nm)				Red (633nm)	
	Band pass filter		448/45	528/45	606/36	755 LP	527/32	586/42	700/54	783/56	660/10	783/56
**Panel**	**Data in**	**Cytometer**										
**cDC2 frequencies in PBMCs**	fig. 1A, 2A,B	Lyric	CD1c BV421	L/D e506		CD45 BV785		CD3/CD56/CD20 PE			CD11c APC	CD14 APC-H7
**cDC2 phenotype HD I**	fig. 1C,D,E, fig. S5	Verse	CD1c BV421	L/D e506			CD20 FITC	CD206 PE	HLA-DR PerCP	MERTK PE-Cy7	CD14 APC	CD11b APC-Cy7
**cDC2 phenotype HD II**	fig. 1C,D,E, fig. S5	Verse	CD1c BV421	L/D e506			CD5 FITC	CD163 PE	CD19 PerCP	PD-L1 PE-Cy7		CD14 APC-H7
**cDC2 phenotype HD III**	fig. 1C,D,E, fig. S5	Verse	CD1c BV421	L/D e506			***IDO AF488***		CD19 PerCP	***IL-10 PE-Cy7***	CD14 APC	
**cDC2 cultured**	fig. 3E,F fig. 4A,C,F fig. 5, fig. 6B,C,E, fig. S3D, S4	Verse	CD1c BV421	L/D e506					HLA-DR PerCP		CD14 APC[a]	CD14 APC-H7[a]
**Purity after isolation**	fig. S3B	Verse	CD1c BV421	L/D e506			CD20 FITC	CD3 PE				CD14 APC-H7
**Sort panel for Aria**	fig. 1E/fig. 3C	Aria	CD1c BV421				CD3/CD56/CD20 FITC					CD14 APC-H7
**Sort panel for Melody**	fig. 1E, fig. 2	Melody					CD3/CD56/CD20 FITC	CD1c PE				CD14 APC-H7
**T cell proliferation panel**	fig. 1E,6D, fig. S2	Lyric	CD4 BV421	CD3 BV510			CFSE			CD25 PE-Cy7	CD8 APC	L/D e780
**Maturation markers HD**	fig. S1G	Verse	CD1c BV421	HLA-DR BV510			HLA-ABC FITC		CD80 PerCP-eFluor710		CD86 APC	CD14 APC-H7
**Maturation markers NSCLC**	fig. S1F	Verse	CD1c BV421	L/D e506			HLA-ABC FITC	CD86 PE	HLA-DR PerCP	CD80 PE-Cy7		CD14 APC-H7
**CD8 NY-ESO1 assay**	fig. 2C,D	Lyric	CTV		CD8 BV605			PD-1 PE		CD25 PE-Cy7		L/D e780
**CD4 NY-ESO1 assay**	fig. 2F,G	Lyric	CTV			CD4 BV786	CD127 FITC			CD25 PE-Cy7		L/D e780
**D7 monocytes DCs I**	fig. S3C	Lyric	CD1c BV421	L/D e506	CD163 BV605		CD206 FITC	CD11b PE	HLA-DR PerCP	PD-L1 PE-Cy7		CD14 APC-H7

**D7 monocytes DCs II**	fig. S3C	Lyric	CD1c BV421	L/D e506	CD163 BV605		CD5 FITC	CD80 PE	CD16 PerCP-Cy5.5	MERTK PE-Cy7		CD14 APC-H7
**PBMC purity +cultured**	fig. 3G,H	Lyric	CD1c BV421	L/D e506	CD163 BV605	CD11c BV785	CD34 FITC	CD3/CD56/CD20 PE				CD14 APC-H7

[a] CD14 APC or CD14 APC-H7 was used

[b] Intracellular staining are italicized and in green

Table S6. Dilutions for the antibodies used in the study and reported in the Key resource table. Related to Figures 1-6.

Marker	Fluorochrome	Clone	Dilution	RRID identifier
CD45	BV785	HI30	1 in 25	(BioLegend Cat# 304048, RRID:AB_2563129)
CD11b	APC cy7	ICRF44	1 in 40	(BioLegend Cat# 301342, RRID:AB_2563395)
CD11c	APC	B-ly6	1 in 20	(BD Biosciences Cat# 559877, RRID:AB_398680)
CD14	APC	M5E2	1 in 40	(BioLegend Cat# 301808, RRID:AB_314190)
CD14	APC-H7	MφP9	1 in 30	(BD Biosciences Cat# 560180, RRID:AB_1645464)
CD163	PE	GHI/61	1 in 25	(BD Biosciences Cat# 556018, RRID:AB_396296)
CD19	PerCP	4G7	1 in 30	(BD Biosciences Cat# 345778, RRID:AB_2868806)
CD1c	BV421	L161	1 in 30	(BioLegend Cat# 331526, RRID:AB_10962909)
CD1c	PE	AD5-8E7	1 in 50	(Miltenyi Biotec Cat# 130-113-302, RRID:AB_2726081)
CD20	FITC	L27	1 in 10	(BD Biosciences Cat# 345792, RRID:AB_2868818)
CD206	PE	19.2(RUO)	1 in 10	(BD Biosciences Cat# 555954, RRID:AB_396250)
CD5	FITC	L17f12	1 in 100	(Thermo Fisher Scientific Cat# 11-0058-42, RRID:AB_1944383)
HLA-DR	PerCP	L243	1 in 30	(BioLegend Cat# 307628, RRID:AB_893566)
HLA-DR	BV510	L243	1 in 20	(BioLegend Cat# 307646, RRID:AB_2561948)
HLA-ABC	FITC	REA230	1 in 25	(Miltenyi Biotec Cat# 130-101-446, RRID:AB_2652080)
CD80	PE-Cy7	L307.4	1 in 20	(BD Biosciences Cat# 561135, RRID:AB_10561688)
CD80	PerCP-eFluor710	2D10.4	1 in 30	(Thermo Fisher Scientific Cat# 46-0809-42, RRID:AB_10548359)
CD86	PE	FUN-1	1 in 20	(BD Biosciences Cat# 555658, RRID:AB_396013)
CD86	APC	FUN-1	1 in 20	(BD Biosciences Cat# 555660, RRID:AB_398608)
IDO1	AF488	#700838	1 in 20	(R and D Systems Cat# IC6030G, RRID:AB_10997134)
IL-10	PE-Cy7	JES3-9D7	1 in 20	(BioLegend Cat# 501420, RRID:AB_2125385)
Mertk	PE-Cy7	590H11G1E3	1 in 30	(BioLegend Cat# 367609, RRID:AB_2687286)
PD-L1	PE-Cy7	MIH1	1 in 20	(BD Biosciences Cat# 558017, RRID:AB_396986)
CD3	FITC	HIT3a	1 in 10	(BD Biosciences Cat# 555339, RRID:AB_395745)
CD56	FITC	NCAM16.2	1 in 10	(BD Biosciences Cat# 345811, RRID:AB_2868832)
CD4	BV421	RPA-T4	1 in 50	(BD Biosciences Cat# 562424, RRID:AB_11154417)
CD8	APC	RPA-T8	1 in 50	(BD Biosciences Cat# 555369, RRID:AB_398595)
CD8	BV605	G42-8	1 in 50	(BD Biosciences Cat# 743066, RRID:AB_2741260)
CD25	PE-Cy7	BC96	1 in 30	(BioLegend Cat# 302612, RRID:AB_314282)
CD3	BV510	SK7	1 in 50	(BioLegend Cat# 344828, RRID:AB_2563704)
CD3	PE	HIT3a	1 in 25	(BD Biosciences Cat# 555340, RRID:AB_395746)
CD56	PE	5.1H11	1 in 25	(BioLegend Cat# 981202, RRID:AB_2715758)
CD20	PE	2H7	1 in 20	(BioLegend Cat# 302306, RRID:AB_314254)
PD-1	PE	MIH4	1 in 25	(BD Biosciences Cat# 557946, RRID:AB_647199)
CD4	BV786	SK3	1 in 50	(BD Biosciences Cat# 563877, RRID:AB_2738462)
CD127	FITC	A019D5	1 in 20	(BioLegend Cat# 351312, RRID:AB_10897643)
CD163	BV605	GHI/61	1 in 20	(BioLegend Cat# 333616, RRID:AB_2616879)
CD206	FITC	19.2	1 in 10	(BD Biosciences Cat# 551135, RRID:AB_394065)
CD11b	PE	ICRF44	1 in 25	(BioLegend Cat# 301306, RRID:AB_314158)

CD80	PE	L307.4	1 in 10	(BD Biosciences Cat# 557227, RRID:AB_396606)
CD16	PerCPCy5.5	3G8	1 in 20	(BD Biosciences Cat# 560717, RRID:AB_1727434)
CD11c	BV785	3.9	1 in 20	(BioLegend Cat# 301644, RRID:AB_2565779)
CD34	FITC	561	1 in 20	(BioLegend Cat# 343604, RRID:AB_1732005)
