# Supplementary material for: Inhibition of CSF-1R and IL-6R prevents conversion of cDC2s into immune incompetent tumor-induced DC3s boosting DC-driven therapy potential
Source: Cell Rep Med. 2024 Jan 18;5(2):101386. doi: 10.1016/j.xcrm.2023.101386 (PMC10897516; doi:10.1016/j.xcrm.2023.101386)
Supplement: Document S2. Article plus supplemental information [file mmc3.pdf]

Cell Reports Medicine

Article

Inhibition of CSF-1R and IL-6R prevents conversion of cDC2s into immune incompetent tumor-induced DC3s boosting DC-driven therapy potential

Graphical abstract

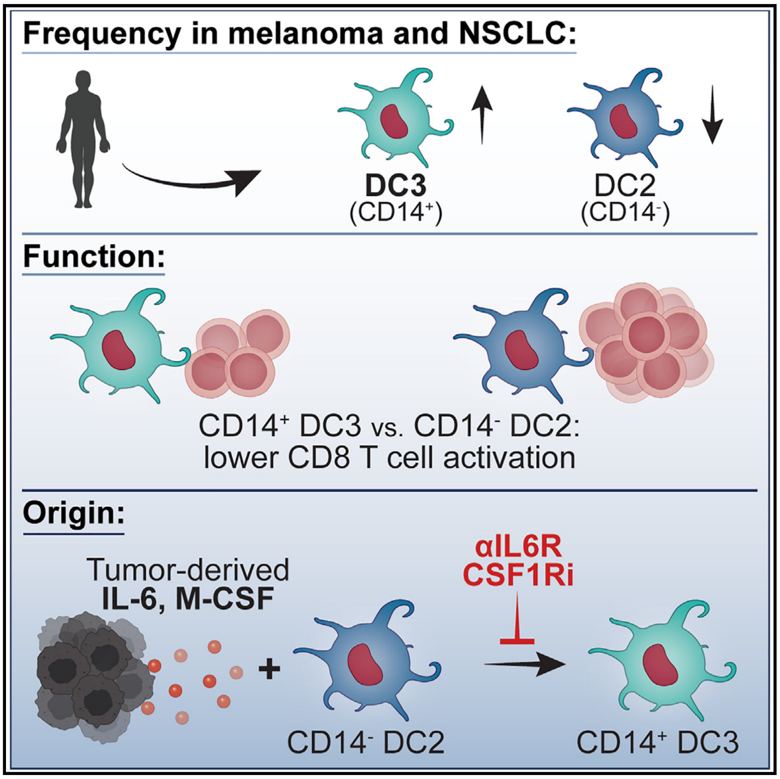

Highlights

- Melanoma and NSCLC patients have increased immune incompetent DC3 frequencies
- Tumor-induced DC3s induce weak tumor antigen-specific CD8 T cell activation
- DC2s, but not monocytes, convert to DC3s driven by tumor-derived M-CSF and IL-6
- CSF1R and IL-6R inhibition prevents tumor-induced DC3s and boosts T cell activation

Authors

Anouk M.D. Becker, Annika H. Decker, Georgina Flórez-Grau, ..., Erik H.J.G. Aarntzen, Martijn Verdoes, I. Jolanda M. de Vries

Correspondence

jolanda.devries@radboudumc.nl

In brief

Becker et al. identify tumor-induced $CD1c^+CD14^+$ DC3s, increased in melanoma and NSCLC patients, originating from DC2s upon exposure to tumor-derived IL-6 and M-CSF. They have an immunosuppressive phenotype and weak T cell activation capacity. Their formation can be prevented by pharmacological inhibition of the IL-6 and M-CSF receptors, rescuing T cell activation capacities.

Becker et al., 2024, Cell Reports Medicine 5, 101386
February 20, 2024 © 2024 The Authors.
https://doi.org/10.1016/j.xcrm.2023.101386

CellPress

Cell Reports Medicine

CellPress
OPEN ACCESS

Article

Inhibition of CSF-1R and IL-6R prevents conversion of cDC2s into immune incompetent tumor-induced DC3s boosting DC-driven therapy potential

Anouk M.D. Becker,[1] Annika H. Decker,[1] Georgina Flórez-Grau,[1] Ghaith Bakdash,[1,7] Rutger J. Röring,[2] Suzan Stelloo,[3] Michiel Vermeulen,[3] Berber Piet,[4] Erik H.J.G. Aarntzen,[5] Martijn Verdoes,[1,6,8] and I. Jolanda M. de Vries[1,8,9,*]

[1]Department of Tumor Immunology, Radboud Institute for Molecular Life Sciences, Radboud University Medical Center, 6525 GA Nijmegen, the Netherlands

[2]Department of Internal Medicine and Radboud Center for Infectious Diseases, Radboud Institute for Molecular Life Sciences, Radboud University Medical Center, 6525 GA Nijmegen, the Netherlands

[3]Department of Molecular Biology, Faculty of Science, Radboud Institute for Molecular Life Sciences, Oncode Institute, Radboud University Nijmegen, 6525 GA Nijmegen, the Netherlands

[4]Department of Pulmonology, Radboud University Medical Center, 6525 GA Nijmegen, the Netherlands

[5]Department of Medical Imaging, Radboud University Medical Center, 6525 GA Nijmegen, the Netherlands

[6]Institute for Chemical Immunology, Radboud Institute for Molecular Life Sciences, Radboud University Medical Center, 6525 GA Nijmegen, the Netherlands

[7]Present address: Microbiotica Ltd, Cambridge, UK

[8]These authors contributed equally

[9]Lead contact

*Correspondence: jolanda.devries@radboudumc.nl

https://doi.org/10.1016/j.xcrm.2023.101386

SUMMARY

The human dendritic cell (DC) family has recently been expanded by CD1c$^+$CD14$^+$CD163$^+$ DCs, introduced as DC3s. DC3s are found in tumors and peripheral blood of cancer patients. Here, we report elevated frequencies of CD14$^+$ cDC2s, which restore to normal frequencies after tumor resection, in non-small cell lung cancer patients. These CD14$^+$ cDC2s phenotypically resemble DC3s and exhibit increased PD-L1, MERTK, IL-10, and IDO expression, consistent with inferior T cell activation ability compared with CD14$^-$ cDC2s. In melanoma patients undergoing CD1c$^+$ DC vaccinations, increased CD1c$^+$CD14$^+$ DC frequencies correlate with reduced survival. We demonstrate conversion of CD5$^{+/-}$CD1c$^+$CD14$^-$ cDC2s to CD14$^+$ cDC2s by tumor-associated factors, whereas monocytes failed to express CD1c under similar conditions. Targeted proteomics identified IL-6 and M-CSF as dominant drivers, and we show that IL-6R and CSF1R inhibition prevents tumor-induced CD14$^+$ cDC2s. Together, this indicates cDC2s as direct pre-cursors of DC3-like CD1c$^+$CD14$^+$ DCs and provides insights into the importance and modulation of CD14$^+$ DC3s in anti-tumor immune responses.

INTRODUCTION

Mononuclear phagocytes (MNPs) are a heterogeneous family of cells, pivotal in bridging innate and adaptive immunity. In human peripheral blood, MNPs comprise monocytes, macrophages, and dendritic cells (DCs). Diverse DC populations have been described over the past decade, accelerated by single-cell technologies.[1] DC subsets are defined by distinct ontogeny, phenotype, transcriptome, and specialized functions.[2,3] The current consensus classifies human blood DCs into plasmacytoid DCs (pDCs), type 1 conventional DCs (cDC1s), and type 2 conventional DCs (cDC2s). Within cDC2s, two transcriptional clusters named DC2s and DC3s have been reported.[1,4] DC2s align transcriptomically with the classical cDC2s, with a profile closer to cDC1s and phenotypically defined as CD1c$^+$CD5$^{+/-}$CD14$^-$CD163$^-$. DC3s are best distinguished as CD1c$^+$CD5$^-$CD14$^+$CD163$^+$ and share phenotypic and transcriptomic properties with monocytes and cDC2s.[4–6]

cDC1s and DC2s arise from a common DC progenitor (CDP), pre-committed to differentiate to cDC1s or cDC2s in human peripheral blood. For DC3s, two studies report data supporting a CDP-independent ontogenetic pathway. Bourdely et al. obtained DC3s from CD34$^+$ hematopoietic stem and progenitor cells (HSPCs) through a CDP-independent differentiation pathway. DC3 differentiation was driven by a bone marrow-derived murine mesenchymal cell line (MS5) expressing human GM-CSF. The *in vitro* obtained DC3s transcriptionally aligned with *in vivo* blood DC3s.[6] In agreement, Cytlak et al. report two distinct developmental trajectories for DC2s and DC3s. DC2s, together with pDCs and cDC1s, followed an IRF8^{high} pathway, while DC3s developed through the monocyte-related IRF8^{low} pathway.[7] Conversely, a CDP-dependent pathway was suggested based

© 2024 The Authors.
This is an open access article under the CC BY license (http://creativecommons.org/licenses/by/4.0/).

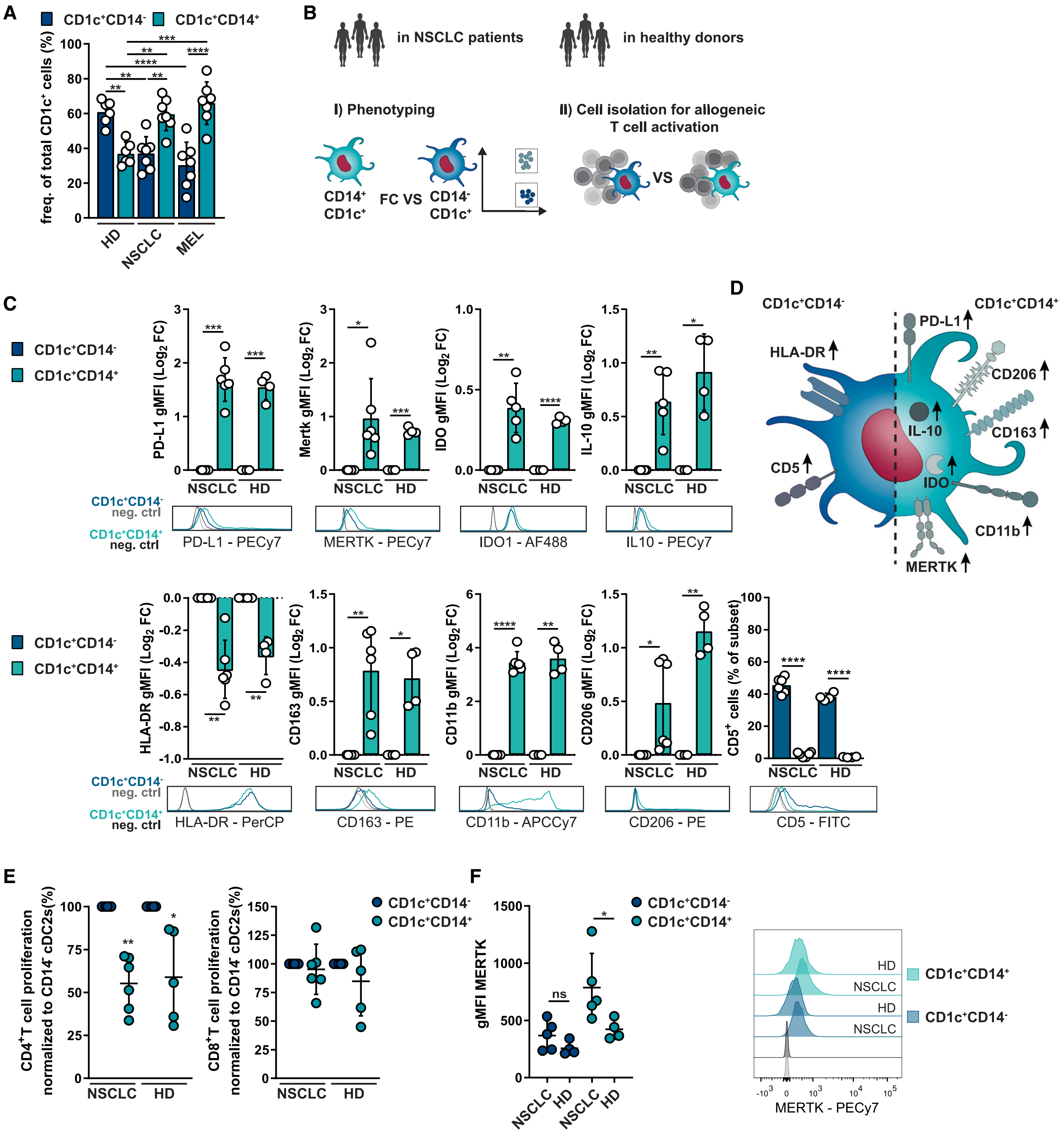

Figure 1. Peripheral blood of lung cancer patients shows increased frequencies of CD1c$^+$CD14$^+$ cells with reduced CD4 T cell activation capability

(A) Ratio of CD14$^+$ and CD14$^-$ cells within CD1c$^+$ cells in peripheral blood of healthy donors (HDs) (n = 6), non-small cell lung cancer (NSCLC) patients (n = 7), and melanoma (MEL) patients (n = 7). Each symbol represents a biological replicate (mean ± SD; one-way ANOVA and Tukey's multiple comparisons test).

(B) Schematic of the assays for (C–F).

(C) Phenotypic hallmarks of freshly isolated CD1c$^+$CD14$^+$ (Log$_2$ fold change [FC] vs. CD1c$^+$CD14$^-$ cells, mean ± SD, paired t test). Each plot is accompanied by a representative histogram.

(D) Summarizing illustration of phenotypic hallmarks.

(legend continued on next page)

on pseudo-time analysis, showing the transition from CD5+ DC2s toward CD163+CD14+ DC3s.[4] Taken together, DC3s can develop following a pathway distinct of the DC2 lineage but whether DC2s contribute to DC3 development remains to be fully dissected.

In a tumor context, another layer of complexity is added to the origin and development of CD1c+CD14+ DCs. The presence of tumor-associated CD1c+CD14+ DCs has been reported in multiple studies including melanoma,[8,9] breast cancer,[6,10] ovarian cancer,[11] and head and neck cancer.[9] In the peripheral blood of stage III and IV melanoma patients, CD1c+CD14+ frequencies were increased compared with healthy individuals.[8] Another study into CD14+ DCs showed that melanoma cells drove cDC2s to CD14+ DCs in a human organotypic skin culture.[12] Although Bourdely et al. did not report the direct effect of tumor cells on DC3 development, a comparison of primary blood DC3s with breast tumor infiltrating CD1c+CD14+ DCs revealed phenotypical and transcriptional alignment.[6] Collectively, these studies emphasize a role for CD1c+CD14+ cells in cancer.

The necessity to study CD14+ DCs in cancer is further highlighted by the impact of defective DCs on immunotherapy efficacy.[13–15] Melanoma patient-derived CD1c+ DC vaccine preparations containing a larger fraction of CD14+ cells significantly hampered T cell activation. Moreover, these CD1c+CD14+ cells exerted antigen-specific CD4 T cell suppression.[8] Strengthening these findings, *in vitro* melanoma-induced CD14+ DCs displayed impaired T cell activation compared with CD14− DCs.[12] Melanoma, as well as non-small cell lung cancer (NSCLC), is characterized by high tumor mutational burden and relatively good response rates to immune checkpoint inhibitors, which revolutionized their treatment.[16–18] Since response rates and efficacy highly vary among patients, is it crucial to consider tumor-induced, impaired DCs as a contributing factor for reduced treatment efficacy in DC-mediated immunotherapies and immune checkpoint inhibitors.[19,20]

Altogether, the tumor microenvironment and associated factors affect developmental, phenotypical, and functional characteristics of DCs. Increased frequencies of DC3s (CD1c+CD14+) are reported in tumor context[6,8–11] but a detailed investigation into their development, characteristics, and plasticity is lacking. To what extent tumor-induced DC3s developmentally belong to the DC2 lineage, monocytic lineage, or follow exclusively a CDP-independent pathway is of specific interest. We hypothesize that investigating their characteristics and plasticity will provide translational possibilities to modulate tumor-induced DC3s and improve anti-tumor responses. To answer these questions, we set out to characterize CD1c+CD14+ cells in NSCLC and investigate their development in the context of melanoma and NSCLC. CD1c+CD14+ phenotypically resembled DC3s and transcriptomically clustered closer to CD14− cDC2s than CD14+ monocytes. Utilizing melanoma and NSCLC cell lines we show that CD1c+CD14+ cells emerge from cDC2s but not monocytes, through IL-6 and macrophage colony-stimulating factor (M-CSF/CSF1) secreted by tumor cells. The environmentally induced transformation can be prevented by pharmacological inhibition of the IL-6 receptor (IL-6R) and CSF1 receptor (CSF1R) simultaneously. This circumvented the enrichment of CD14+ cDC2s and improved the ability of cDC2s to stimulate T cell proliferation. Our work highlights the plasticity of DCs, provides insights in the development of DC3-like CD1c+CD14+ DCs with cDC2s as pre-cursor, and possibilities to modulate DCs for enhanced anti-tumor responses to improve cancer therapies outcomes.

RESULTS

Peripheral blood of lung cancer patients shows increased frequencies of CD1c+CD14+ cells with reduced CD4 T cell activation capability

We first determined the balance between CD1c+CD14− cells and CD1c+CD14+ cells in peripheral blood of early-stage NSCLC and stage III and IV melanoma patients by flow cytometry and compared this with healthy donors (HDs). This revealed an altered balance within the CD1c+ fraction (Figure 1A). In NSCLC and melanoma patients, the majority of CD1c+ cells, on average 59% and 68%, respectively, expresses CD14. In contrast, in HDs, a minority (38%) of CD1c+ cells is characterized as CD1c+CD14+. Herewith, we add enrichment of CD1c+CD14+ myeloid cells in NSCLC patients to the previous observations in peripheral blood of metastatic melanoma and several tumor tissues[6,8–10] (Figure S1A). For melanoma, we analyzed patients receiving CD1c+ DC-based vaccinations in a clinical trial performed by our group[20] to study the clinical relevance of increased CD1c+CD14+ cells. The fraction of CD1c+CD14+ cells in peripheral blood and the manufactured autologous DC vaccines varied between patients (Figures S1C and S1D). Analysis of correlation between CD1c+CD14+ frequencies and progression-free survival (PFS) for responding melanoma patients showed that high CD1c+CD14+ frequencies in the vaccine correlate with shorter PFS ($R^2 = 0.9570$, $p = 0.0038$, $n = 5$) (Figure S1B). After each DC vaccination, PBMCs are tested for the presence of KLH-specific T cells for immunomonitoring purposes.[20] Analysis of correlation between CD1c+CD14+ frequencies in PBMCs with KLH-specific T cells showed an increase in proliferation index with decreasing CD14+ cDC2 frequencies ($R^2 = 0.4898$, $p = 0.0165$), and a similar trend was observed for CD14+ cDC2 frequencies in the vaccines (Figures S1D and S1E).

For NSCLC patients we performed flow cytometric analysis to phenotypically characterize the CD1c+CD14+ cells. CD1c+CD14+ cells display a macrophage-like phenotype with higher expression of CD163, CD206, and MER proto-oncogene tyrosine kinase (MERTK) compared with CD1c+CD14− cells (Figures 1B–1D). The largest differences were observed for the monocytic marker CD11b, where CD1c+CD14+ cells show a 3.45–3.60 $\log_2$ fold increase in expression. CD5, a pre-DC-related marker defining DC2s and absent on DC3s,[4,5] was expressed by 38%–45% of CD1c+CD14− cells and absent on CD1c+CD14+ cells

(E) Allogeneic CD4 and CD8 T cell proliferation after 5-day co-culture with immature CD1c+CD14− or CD1c+CD14+ cells (mean ± SD, paired t test). Biological replicates were n = 6 for NSCLC patients, n = 5 for HDs, each one consisting of technical duplicates.

(F) MERTK expression differences between NSCLC patients and HDs accompanied by representative histograms (mean ± SD, two-way ANOVA, Sidak's multiple comparison test). For (C) and (F), n = 4 biological replicates for HDs and n = 6 for NSCLC patients. gMFI, geometric mean fluorescent intensity *p < 0.05, **p < 0.01. See also Figure S1.

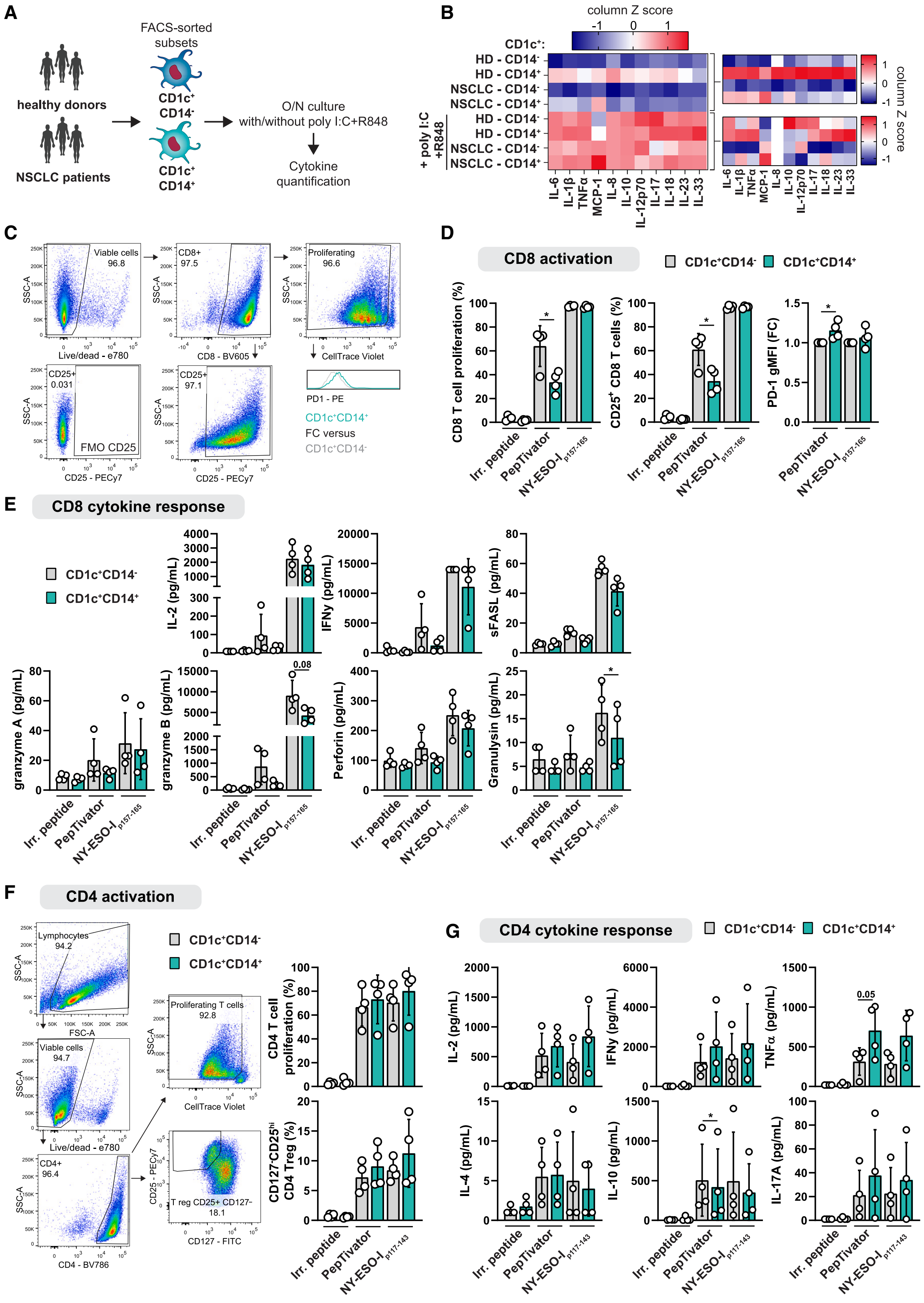

(legend on next page)

CellPress
OPEN ACCESS

(Figure 1C). To gain insight into their immunosuppressive profile, we measured programmed death ligand 1 (PD-L1), and intracellular IL-10 and indoleamine 2,3-dioxygenase (IDO) expression. All markers were significantly higher in CD1c$^+$CD14$^+$ cells compared with CD1c$^+$CD14$^-$ cells, consistent for HDs and NSCLC patients (Figure 1C). The major histocompatibility complex class II (MHC class II) receptor HLA-DR was strongly reduced on freshly isolated CD1c$^+$CD14$^+$ cells (Figure 1C) as well as on cultured CD1c$^+$CD14$^+$ cells (Figures S1F and S1G), in line with an almost 50% reduction of allogeneic CD4 T cell activation (Figure 1E). Both subsets induced similar levels of allogeneic CD8 T cell activation, in agreement with similar MHC class I receptor HLA-ABC expression (Figures 1E, S1F, and S1G).

Strikingly, MERTK, known to be involved in immunotolerance and activating oncogenic signaling pathways,[21,22] was the only protein significantly higher on CD1c$^+$CD14$^+$ cells of NSCLC patients compared with HDs (Figure 1F). Taken together, CD1c$^+$CD14$^+$ cells from HDs and NSCLC patients show high phenotypic and functional resemblance, with macrophage-like and immunosuppressive characteristics.

CD1c$^+$CD14$^+$ cells produce high amounts of pro-inflammatory cytokines but weak tumor antigen-specific CD8 T cell responses

To further investigate the functional features of CD1c$^+$CD14$^+$ cells in NSCLC patients and HDs, we analyzed their cytokine production using the LEGENDplex Human Inflammation panel (Figure 2A). DCs produced detectable levels of IL-6, IL-1β, TNF-α, MCP1, IL-8, IL-10, IL-12p70, IL-17a, IL-18, IL-23, and IL-33, but IFN-α and IFN-γ were below the detection limit (Figure 2B; Table S3). Immature CD1c$^+$CD14$^+$ cells from HDs displayed a clear pro-inflammatory cytokine profile with high production of all cytokines detected compared with CD1c$^+$CD14$^-$ cells. Although CD1c$^+$CD14$^+$ cells from NSCLC patients produced relatively higher amounts of most cytokines than their CD14$^-$ counterparts, the overall cytokine production by cells of NSCLC patients was low compared with cells of HDs. As expected, stimulation with poly(I:C) and R848 increased the cytokine production and revealed higher amounts of the classical cDC2s cytokine IL-12p70 by CD1c$^+$CD14$^-$ compared with CD1c$^+$CD14$^+$ cells (Figure 2B).

Next, after having observed the weak T cell activation by CD1c$^+$CD14$^+$ cells in an allogeneic setup we set out to assess their ability to stimulate T cells in antigen-specific assays. Tetanus toxoid and cytomegalovirus, pathogens that are encountered by a large fraction of the population (either through infection or vaccination), were utilized to induce antigen-specific recall response of memory T cells. CD1c$^+$CD14$^-$ and CD1c$^+$CD14$^+$ cells induced comparable amounts of autologous antigen-specific CD4 T cell proliferation and slightly lower CD8 T cell proliferation, which did not reach statistical significance (Figures S2A and S2B).

To directly test the ability of CD1c$^+$CD14$^+$ cells to induce anti-tumor CD8 T cell responses, we performed 4-day co-cultures of flow cytometry-sorted CD1c$^+$CD14$^+$ and CD1c$^+$CD14$^-$ cells with autologous CD8 T cells transfected with a T cell receptor (TCR) that recognizes the NY-ESO1$_{p157\text{-}165}$ peptide, derived from tumor antigen New York esophageal squamous cell carcinoma 1 (NY-ESO1), presented in HLA-A*02:01 (Figure 2C). CD1c$^+$CD14$^+$ and CD1c$^+$CD14$^-$ cells treated with the short NY-ESO1$_{p157\text{-}165}$ peptide performed equally well with regard to CD8 T cell proliferation and activation (Figure 2D). Interestingly, treatment with a mixture of longer (15-mer) NY-ESO1 peptides, called PepTivator, resulted in a strong reduction of CD8 T cell proliferation and activation from 64% in co-cultures with CD1c$^+$CD14$^-$ cells to 34% with CD1c$^+$CD14$^+$ cells possibly related to a weaker ability to cross-present antigens (Figure 2D). Moreover, CD8 T cells activated by CD1c$^+$CD14$^+$ cells expressed higher levels of the regulatory immune checkpoint programmed cell death protein 1 (Figure 2D) and produced lower amounts of granulysin and granzyme B, suggesting weaker cytotoxic activity of the induced CD8 T cells (Figure 2E).

In a similar setup using autologous CD4 T cells transfected with TCR specific for the NY-ESO1$_{p117\text{-}143}$ peptide presented in HLA-DRB1*04:01, CD1c$^+$CD14$^+$ and CD1c$^+$CD14$^-$ cells showed similar CD4 T cell stimulatory capacity (Figure 2F). Assessment of T cell effector differentiation showed high inter-individual variation and no large differences in the production of IL-2, IL-4, IFN-γ, IL-10, TNF-α, or IL-17A by T cells activated by CD1c$^+$CD14$^+$ compared with CD1c$^+$CD14$^-$ cells (Figure 2G).

Altogether, we observed that HD CD1c$^+$CD14$^+$ cells are capable of producing large amounts of cytokines, but these cells are strongly affected in NSCLC patients. Moreover, CD1c$^+$CD14$^+$ cells show a defective stimulatory capacity of tumor antigen-specific CD8 T cell responses compared with CD1c$^+$CD14$^-$ cells.

cDC2s, but not monocytes, transdifferentiate into CD1c$^+$CD14$^+$ cells in response to tumor cues

The increased frequencies and low immunostimulatory capacity of CD1c$^+$CD14$^+$ cells urged us to study their development in

Figure 2. CD1c$^+$CD14$^+$ cells produce high amounts of pro-inflammatory cytokines, but weak tumor antigen-specific CD8 T cell responses

(A) CD1c$^+$CD14$^-$ and CD1c$^+$CD14$^+$ cells from HDs (n = 5) and NSCLC patients (n = 6) were FACS sorted and cultured overnight with or without poly(I:C) (20 μg/mL) and R848 (4 μg/mL). Cytokine quantification in supernatants was performed using the LEGENDplex Human Inflammation panel.

(B) Heatmap displaying *Z* scores calculated on log-transformed data of the average cytokine concentrations for all conditions (B, left) and separately for unstimulated and stimulated conditions (B, right), with n = 5 biological replicates for HDs and n = 6 for NSCLC patients. See also Table S3.

(C and D) (C) Representative dot plots and PD-1 histogram showing gating strategy to analyze the activation of CD8 T cells (D) transfected with NY-ESO1-specific TCR, by autologous FACS-sorted CD1c$^+$CD14$^-$ and CD1c$^+$CD14$^+$ cells from HLA-A*02:01$^+$ HDs that were pre-treated with irrelevant (Irr.) peptide, PepTivator NY-ESO1 (mainly 15-mer peptide mix), or NY-ESO1$_{p157\text{-}165}$ peptide.

(E) Cytokines secreted in culture media after 24 h of co-culture.

(F) Gating strategy, CD4 T cell proliferation, and frequency of regulatory CD4 T cells of CD4 T cells transfected with NY-ESO1-specific TCR, activated by autologous FACS-sorted CD1c$^+$CD14$^-$ and CD1c$^+$CD14$^+$ cells from HLA-DRB1*04:01$^+$ HDs (n = 4) that were pre-treated with irrelevant (Irr.) peptide, PepTivator NY-ESO1 (mainly 15-mer peptide mix), or NY-ESO1$_{p117\text{-}143}$ peptide.

(G) Cytokines secreted in culture medium after 24 h of co-culture. (D–G) Symbols depict individual donors, with n = 4 biological replicates for all experiments, each the mean of technical triplicates (mean ± SD, repeated measures [RM] one-way ANOVA with Sidak's multiple comparisons test) *p < 0.05 See also Figure S2.

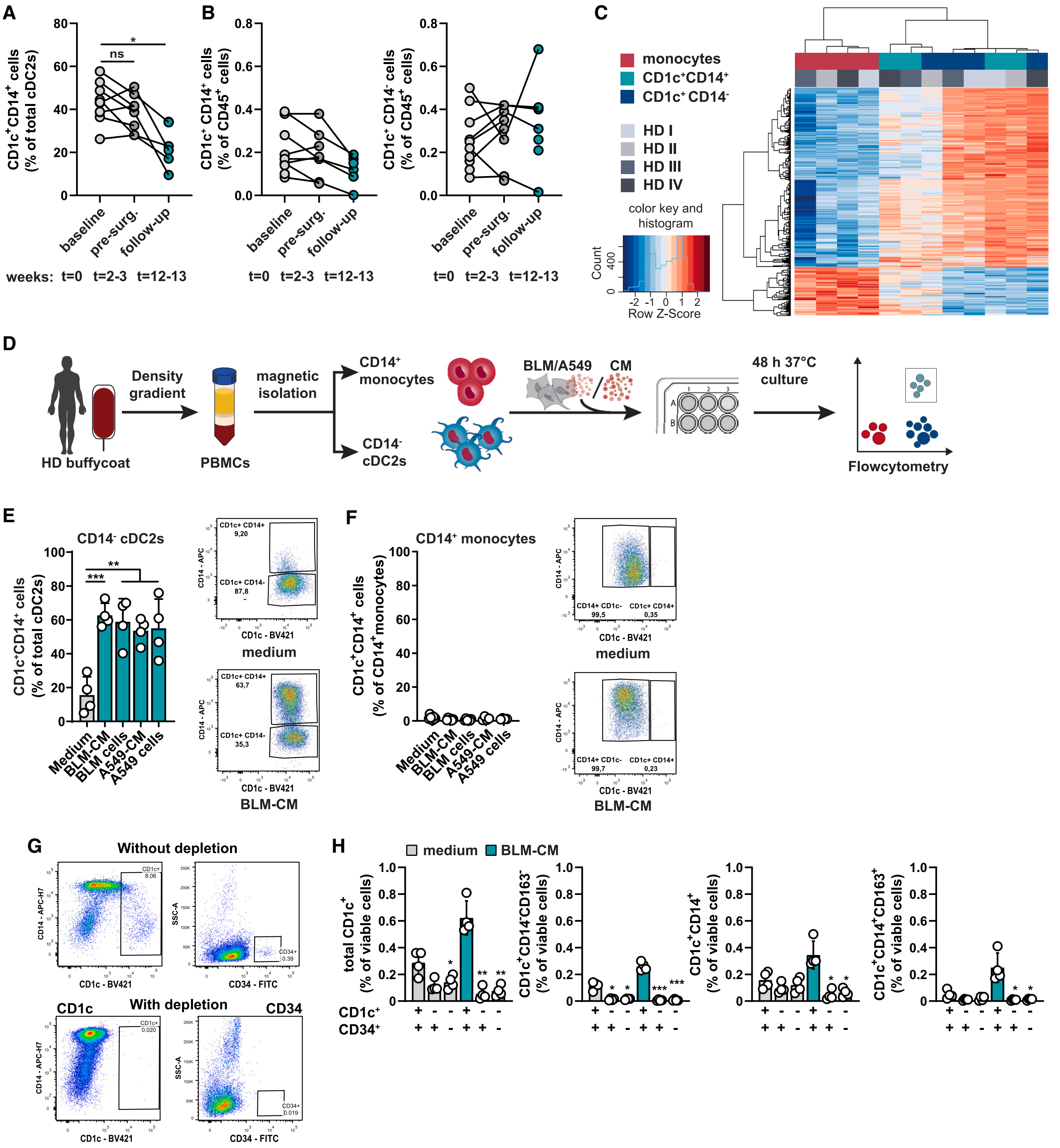

Figure 3. cDC2s, but not monocytes, transdifferentiate into CD1c^{+}CD14^{+} cells in response to tumor cues

(A and B) Frequency of CD1c^{+}CD14^{+} cells expressed as fraction of total cDC2s (A) and total CD45^{+} cells (B) in peripheral blood of NSCLC patients over the course of treatment in the DONAN trial. Baseline, prior to Durvalumab (anti-PD-L1) treatment (t = 0); pre-surg., after two courses of Durvalumab before surgery for NSCLC tumor resection (t = week 2–3) and follow-up 10 weeks after surgery (t = week 12–13). Each symbol represents a patient, lines connect different time points from the same patient (mixed effects analysis, Dunnett's multiple comparisons test), with n = 10, 8, and 7 biological replicates for the different time points, respectively.

(C) Heatmap displaying DEGs between CD1c^{+}CD14^{-} cells vs. CD14^{+} monocytes and CD1c^{+}CD14^{+} cells vs. CD14^{+} monocytes, as analyzed by RNA Affymetrix Array Eurofins. Number of probes used for hierarchical clustering in heatmap is 387, n = 4 biological replicates.

(legend continued on next page)

tumor context. The NSCLC patients were part of a clinical trial (NCT03853187) in which two courses of anti-PD-L1 monoclonal antibody were administered prior to scheduled curative resection of the primary tumor. Assessment of $CD1c^+CD14^+$ frequencies in their PBMCs upon inclusion (baseline), after anti-PD-L1 treatment but before surgery, and 10 weeks after surgery, enabled us to follow $CD1c^+CD14^+$ cell frequencies over time in NSCLC patients. Whereas anti-PD-L1 treatment had no significant effect, removal of the tumor resulted in a significant reduction of $CD1c^+CD14^+$ cells back to the levels of HDs (Figures 1A and 3A). Analysis of frequencies of total $CD45^+$ cells shows that $CD1c^+CD14^-$ frequencies remain similar and a loss in $CD1c^+CD14^+$ cells restored the balance (Figure 3B).

After having observed the influence of the tumor on $CD1c^+CD14^+$ cell frequencies, we continued with investigating potential precursors of tumor-induced $CD1c^+CD14^+$ cells. Considering that several studies showed that $CD1c^+CD14^+$ cells share similarities with $CD1c^+$ DCs (cDC2s) and $CD14^+$ monocytes,[8,11,23,24] we started with cDC2s or $CD14^+$ monocytes as potential precursors for transcriptomic analysis and *in vitro* differentiation assays. To compare transcriptomics, RNA was isolated from FACS-sorted $CD1c^+CD14^-$ DCs, $CD1c^+CD14^+$ cells. and $CD14^+$ monocytes from blood of four HDs and analyzed by Affymetrix microarrays. Analysis of differentially expressed genes (DEGs) did not retrieve any DEGs (Log_2 fold change > 1.5) between $CD1c^+CD14^-$ and $CD1c^+CD14^+$ cells after correction for multiple testing (Figure S3A). Subsequent hierarchical clustering of DEGs across all three populations showed $CD1c^+CD14^+$ cells clustering closer to $CD1c^+CD14^-$ DCs, supporting that $CD1c^+CD14^+$ cells are closer related to cDC2s than to $CD14^+$ monocytes on the transcriptomic level (Figure 3C).

To assess whether DCs and monocytes can give rise to tumor-induced $CD1c^+CD14^+$, we started *in vitro* differentiation assays with $CD14^-$ cDC2s or $CD14^+$ monocytes isolated from HD PBMCs (purity $\geq$ 95%, Figure S3B). We used tumor cell line co-cultures or mono-cultures in medium conditioned by these tumor cell lines as a uniform model to examine involved tumor-associated factors (Figure 3D). BLM and A549 cells were used to mimic melanoma and NSCLC tumor cues, respectively. Two-day co-cultures of cDC2s with BLM or A549 cells led to a clear subpopulation of 59% and 55% $CD1c^+CD14^+$ cells, respectively (Figure 3E). Monoculture in tumor-conditioned medium (CM) caused a similar increase in the amount of $CD1c^+CD14^+$ cells (63% and 54%) as direct tumor-cell contact, whereas cDC2s cultured in medium led to 16% $CD14^+$ cells. This suggests that soluble factors are sufficient to induce a majority of $CD1c^+CD14^+$ cells. Independent of the cancer cell line used, monocytes failed to upregulate CD1c and only showed a small increase in CD14 expression in response to cancer cell line-derived cues (Figure 3F). To assess if the emergence of $CD1c^+CD14^+$ cells is solely dependent on $CD1c^+CD14^-$ cells, we depleted PBMCs from all $CD1c^+$ cells prior to a 2-day culture with BLM-CM. As expected, BLM-CM increased the frequency of $CD1c^+CD14^+$ cells in PBMC cultures, which was largely prevented by CD1c depletion (Figures 3G and 3H). A small fraction of $CD1c^+CD14^+$ cells was detectable, which was not derived from $CD34^+$ progenitor cells as PBMCs depleted for both CD1c and CD34 resulted in similar frequencies. Interestingly, inclusion of the DC3 marker CD163 showed that the $CD1c^+CD14^+$ fraction is $CD163^-$ and no $CD1c^+CD14^+CD163^+$ cells arose when all $CD1c^+$ cells were depleted (Figure 3H).

Next, a characterization of $CD1c^+CD14^+$ cells after a 7-day culture period showed that long culture periods induce a phenotype in $CD1c^+CD14^+$ cells that largely resembles monocytes/macrophages, with high expression of CD14, CD206, CD11b, and CD163 while CD1c surface expression was lost in both medium and BLM-CM conditions (Figure S3C). Finally, based on the observed systemic effect of tumors on $CD1c^+CD14^+$ cell frequencies in patients, we cultured $CD14^-$ cDC2s with melanoma patient or HD serum. Culture with HD serum showed that a fraction of cDC2s upregulates CD14, which slightly increases with melanoma patient serum (Figure S3D). In conclusion, we establish cDC2s and not monocytes as potential precursors of tumor-induced $CD1c^+CD14^+$ cells, and hence refer to $CD1c^+CD14^-$ cells as cDC2s and $CD1c^+CD14^+$ cells as $CD14^+$ cDC2s. These data furthermore support a shared lineage for DC3s ($CD1c^+CD14^+$) with DC2s,[4] suggesting that DC3s can develop from a CDP.

Tumor-associated proteins M-CSF and IL-6 drive transdifferentiation toward $CD14^+$ cDC2s

Next, we set out to investigate the cancer-associated factors involved in the differentiation of cDC2s to $CD14^+$ cDC2s. Culturing cDC2s in the presence of BLM/A549-CM induced $CD14^+$ cDC2s, indicating that soluble factors are involved, and that cell-cell contact is not crucial. This finding was further supported by trans-well experiments where BLM cells placed in a separate and same compartment initiated CD14 expression on cDC2s (Figure S4A). The stronger increase in $CD14^+$ cells with BLM cells in closer proximity suggests that either high local concentrations of soluble molecules or surface molecules enhance the $CD14^+$ cDC2s induction. Considering the high frequencies of up to 63% $CD14^+$ cDC2s after mono-culturing with CM, we hypothesized that soluble factors have the largest impact and therefore continued with attempting to identify those.

A key process through which tumors influence biological processes is the cancer secretome.[25] To establish whether the soluble factors affecting cDC2s are proteins, BLM-CM was heated to 95°C for 10 min to denature proteins. Whereas untreated BLM-CM induced 48% $CD14^+$ cDC2s, heated BLM-CM did not increase the percentages of $CD14^+$ cells compared with medium (Figure S4B). Thus, we performed a targeted proteomics analysis using OLINK Target 96 Immuno-Oncology Panel (OLINK, Uppsala, Sweden). First, several cancer cell lines were

(D–F) (D) Schematics of the assays for (E and F) in which the induction of $CD1c^+CD14^+$ cells is analyzed after a 2-day co-culture of $CD14^-$ cDC2s (E) or $CD14^+$ monocytes (F) from HDs with medium, melanoma BLM conditioned medium (CM), BLM cells, lung cancer A549-CM, or A549 cells. Each symbol represents an individual cDC2 (E) or monocyte (F) donor, performed for n = 4 biological replicates (mean ± SD; one-way ANOVA and Dunnett's multiple comparisons test).
(G and H) (G) Representative flow cytometry dot plots before and after depletion of CD1c and CD34 from PBMCs of HDs, which were subsequently cultured for 2 days in medium or 50% BLM-CM (H). Each symbol represents a biological replicate (n = 4) (mean ± SD), asterisks depict significance vs. medium or BLM-CM (one-way RM ANOVA with Dunnett's multiple comparisons test). *$p < 0.05$, **$p < 0.01$, ***$p < 0.001$. See also Figure S3.

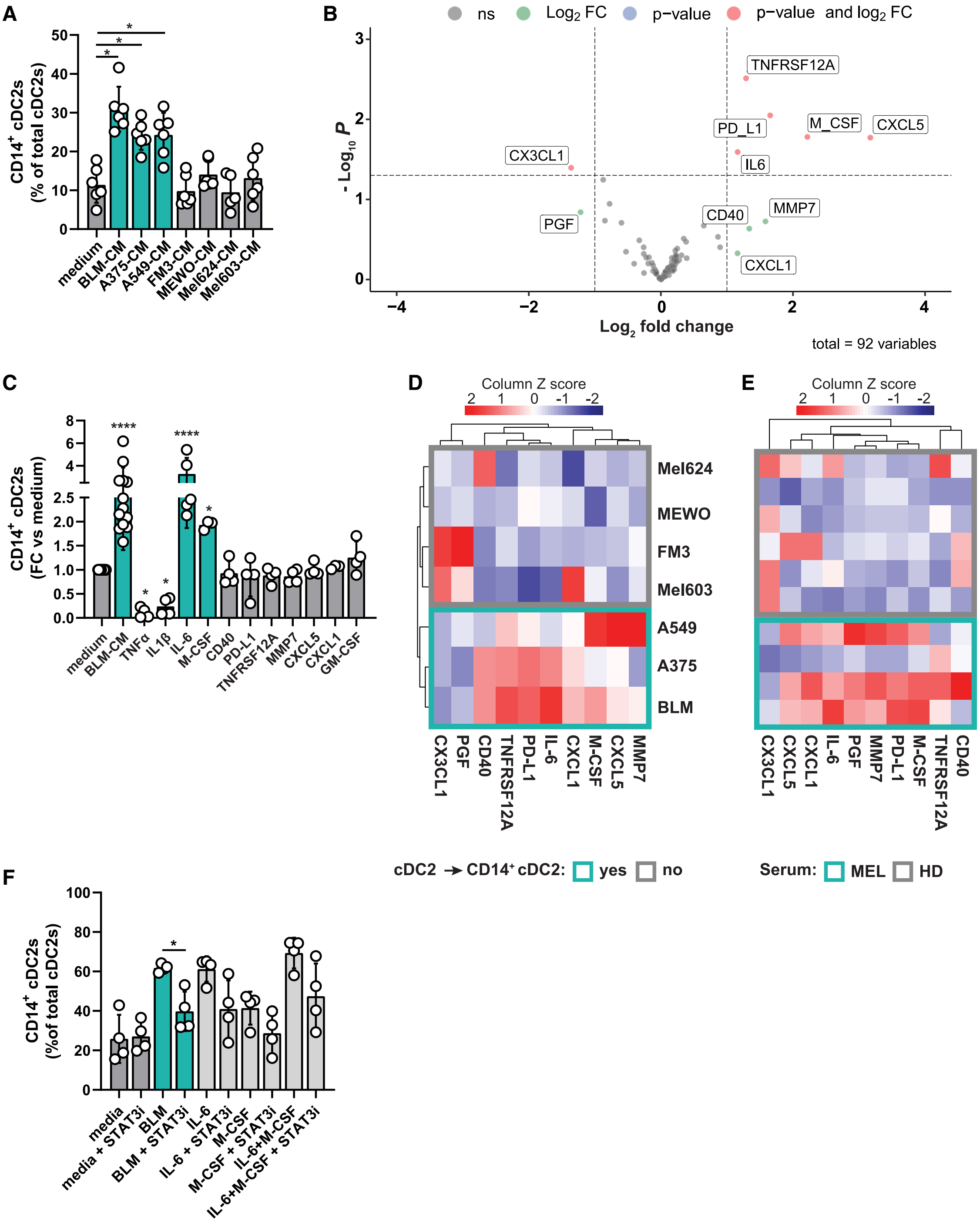

Figure 4. Tumor-associated proteins M-CSF and IL-6 drive transdifferentiation toward CD14+ cDC2s

(A) Screening of 24 h serum-free CM of several cancer cell lines for their ability to induce CD14+ cDC2s from HD, each symbol depicts a biological replicate (mean ± SD, n = 6) (mixed effects analysis, Dunnett's multiple comparisons test).

(B) Volcano plot displaying the log_2 fold change against –log 10 statistical p value for 92 proteins from the OLINK Immuno-Oncology Panel, with n = 3 (BLM, A375, A549) over n = 4 (FM3, MEWO, Mel624, Mel603) different cell lines.

(C) Effect of 2-day culture treatment with recombinant human cytokines on CD14+ cDC2 induction compared with medium. Each symbol represents a biological replicate (mean ± SD), asterisks show significant results compared with medium (mixed effects analysis, Fisher's LSD).

(D and E) Heatmaps showing scaled NPX values of all proteins differentially expressed between BLM/A375/A549-CM (n = 3) and the CM of control cancer cell lines (n = 4) in all individual CM is depicted in (D) and for melanoma (MEL) and healthy donor (HD) serum (without row clustering) in (E), with each heatmap cell showing a biological replicate (n = 4 for MEL, n = 6 for HD).

(legend continued on next page)

CellPress
OPEN ACCESS

tested for their ability to induce CD14$^+$ cDC2s. Of these, BLM, A549, and A375 cells induced CD14$^+$ cDC2s above background frequencies, whereas FM3, MeWo, Mel-624, and Mel603 did not increase CD14 expression and hence served as negative control cell lines (Figure 4A). Next, 24 h serum-free CM from these cell lines was screened for 92 proteins with the OLINK Immuno-Oncology Panel resulting in 8 differentially secreted proteins (Figure 4B). Each of these candidates, TNFRSF12A, PD-L1, M-CSF, CXCL5, IL-6, CD40, MMP7, and CXCL1, were subsequently added to *in vitro* CD14$^-$ cDC2 cultures to assess their capability to induce CD14$^+$ cDC2s from CD14$^-$ cDC2s. Of all eight recombinant proteins only IL-6 and M-CSF induced a significant increase in CD14$^+$ cDC2s with fold changes vs. medium of 3.3 and 2.0, respectively (Figure 4C). Analysis of their relative amounts in CM shows IL-6 abundance in A375 and BLM-CM, and lower amounts in A549-CM that seem compensated by higher M-CSF amounts (Figure 4D). Analysis of the eight candidate proteins in melanoma serum samples showed that three out of four sera contained high levels of M-CSF and IL-6 compared with HD serum (Figure 4E). The recombinant cytokines TNF-α and IL-1β, part of the standard DC maturation cocktail (IL-6, TNF-α, IL-1β, PGE2),[26] were also tested in the *in vitro* assay. Interestingly, TNF-α and IL-1β showed a preventive effect. Where on average 37% of cDC2s in medium expressed CD14 after 2 days, this reduced to 4% and 10% after culture in the presence of TNF-α and IL-1β, respectively (Figure 4C). GM-CSF, known to promote DC development *in vitro*,[27] did not convert cDC2s to CD14$^+$ cDC2s. Finally, treatment of cDC2s with the STAT3 inhibitor Stattic or Janus kinase inhibitor Tofacitinib prior to exposure to BLM-CM, IL-6, or M-CSF showed that both STAT3 and JAK are involved in the downstream signaling of the BLM-CM- and IL-6-driven CD14$^+$ cDC2s (Figures 4F and S4C). Altogether, the transdifferentiation from cDC2s to CD14$^+$ cDC2s is dominantly driven by M-CSF and IL-6, secreted by tumor cells and present in serum from cancer patients.

Healthy cDC2s have phenotypic plasticity and once matured do not convert to CD14$^+$ cDC2s

CD14$^+$ cDC2s negatively affected DC vaccination efficiency[8] and showed reduced CD4 T cell activation in cancer patients. To overcome immune hampering effects of CD14$^+$ cDC2s, opportunities lie in interfering with their development. To be able to intervene, understanding the plasticity of the conversion to CD14$^+$ cDC2s is essential. Since the pro-inflammatory cytokines TNF-α and IL-1β showed a preventive effect, we cultured CD14$^-$ cDC2s with TNF-α and IL-1β, and simultaneously challenged with BLM-CM (Figure 5A). Apparent from the very low frequency of CD14$^+$ cDC2s after a 2-day culture, the effect of TNF-α and IL-1β is strong enough to prevent CD14$^+$ cDC2 formation even in the presence of BLM-CM. The additionally tested GM-CSF and TLR ligands poly(I:C) and R848, showed comparable results in prohibiting conversion to CD14$^+$ cDC2s (Figure 5B).

Since TNF-α, IL-1β, GM-CSF, poly(I:C), and R848 are known stimuli to induce DC maturation, we hypothesized that mature cDC2s are no longer susceptible for conversion to CD14$^+$ cDC2s. To test this, we matured CD14$^-$ cDC2s by pre-treatment with GM-CSF and TLR ligands, followed by culture with BLM-CM for 2 additional days. No differences in cDC2 frequencies were observed between culture with and without BLM-CM when cDC2s were matured with TLR ligands (Figure 5C). Among GM-CSF-matured CD14$^-$ cDC2s, a small frequency of CD14$^+$ cDC2s was induced by BLM-CM (20%), although still minor compared with the increase for immature CD14$^-$ cDC2s (66%).

Finally, to assess the reversibility of the cDC2 transdifferentiation we isolated CD14$^+$ cDC2s from HDs or induced CD14 expression on cDC2s through culture with BLM-CM, both followed by exposure to TNF-α and IL-1β. TNF-α and IL-1β managed to partly revert healthy CD14$^+$ cDC2s to cDC2s, evident from the decreased frequency at day 1 compared with starting material (Figure 5D). Of note, increasing cytokine concentrations did not further reduce the CD14$^+$ fraction (Figures S4D and S4E). In contrast, none of the BLM-CM-induced CD14$^+$ cDC2s lost CD14 expression (Figure 5E), suggesting that contact with tumor-secreted factors alters the plasticity, thereby prohibiting the reversion of CD14$^+$ DC2s to CD14$^-$ cDC2s. Collectively, immature CD14$^+$ cDC2s are phenotypically plastic until they encounter tumor-associated factors that rigidify their plasticity.

Modulation of cDC2s with anti-IL-6R and CSF1Ri prevents CD14$^+$ cDC2s and enhances T cell activation

Focusing on preventing tumor-induced CD14$^+$ cDC2s, we investigated licensed drugs for their ability to impede CD14$^+$ cDC2s. Given the toxicity of repeated systemic administration of pro-inflammatory cytokines,[28] we considered inhibitors of the IL-6R and M-CSF receptor (CSF1R). Tocilizumab, a humanized anti-IL-6R monoclonal antibody, prevents binding of IL-6 to its receptors thereby blocking IL-6 signaling. The US Food and Drug Administration (FDA) authorized tocilizumab for several arthritis treatments, cytokine release syndrome, and COVID-19. To interrupt M-CSF signaling we selected sunitinib, a small-molecule receptor tyrosine kinase inhibitor that is US FDA approved for pancreatic cancer, gastrointestinal stromal tumors, and renal cell carcinoma. To validate the inhibition of IL-6 and M-CSF signaling by tocilizumab and sunitinib, CD14$^-$ cDC2s were cultured for 2 days with IL-6 or M-CSF accompanied by the respective inhibitor (Figure 6A). Drug treatment prevented IL-6- and M-CSF-induced CD14 upregulation resulting in expression levels equal to those observed for control conditions, confirming specificity of the drugs (Figure 6B). Next, CD14$^-$ cDC2s were cultured with BLM-CM to induce CD14$^+$ cDC2s in the absence or presence of anti-IL-6R, CSF1R inhibitor (CSF1Ri), or both. A small reduction in CD14$^+$ cDC2s was observed with singular treatment. Combined inhibition of IL-6 and M-CSF resulted in complete prevention of BLM-CM-induced CD14$^+$ cDC2s, supporting the dominant role of IL-6 and M-CSF in the CD14$^+$

(F) Induction of CD14$^+$ cDC2s in the absence or presence of 1 μM STAT3 inhibitor (STAT3i) Stattic after a 2-day culture period with 50% BLM-CM, 25 ng/mL IL-6 and/or 25 ng/mL M-CSF. Each symbol represents an individual donor, with n = 4 biological replicates (mean ± SD, one-way RM ANOVA with Dunnett's multiple comparisons test between untreated and STAT3i per condition). *p < 0.05, **p < 0.01, ***p < 0.001, ****p < 0.0001. See also Figures S4A–S4C.

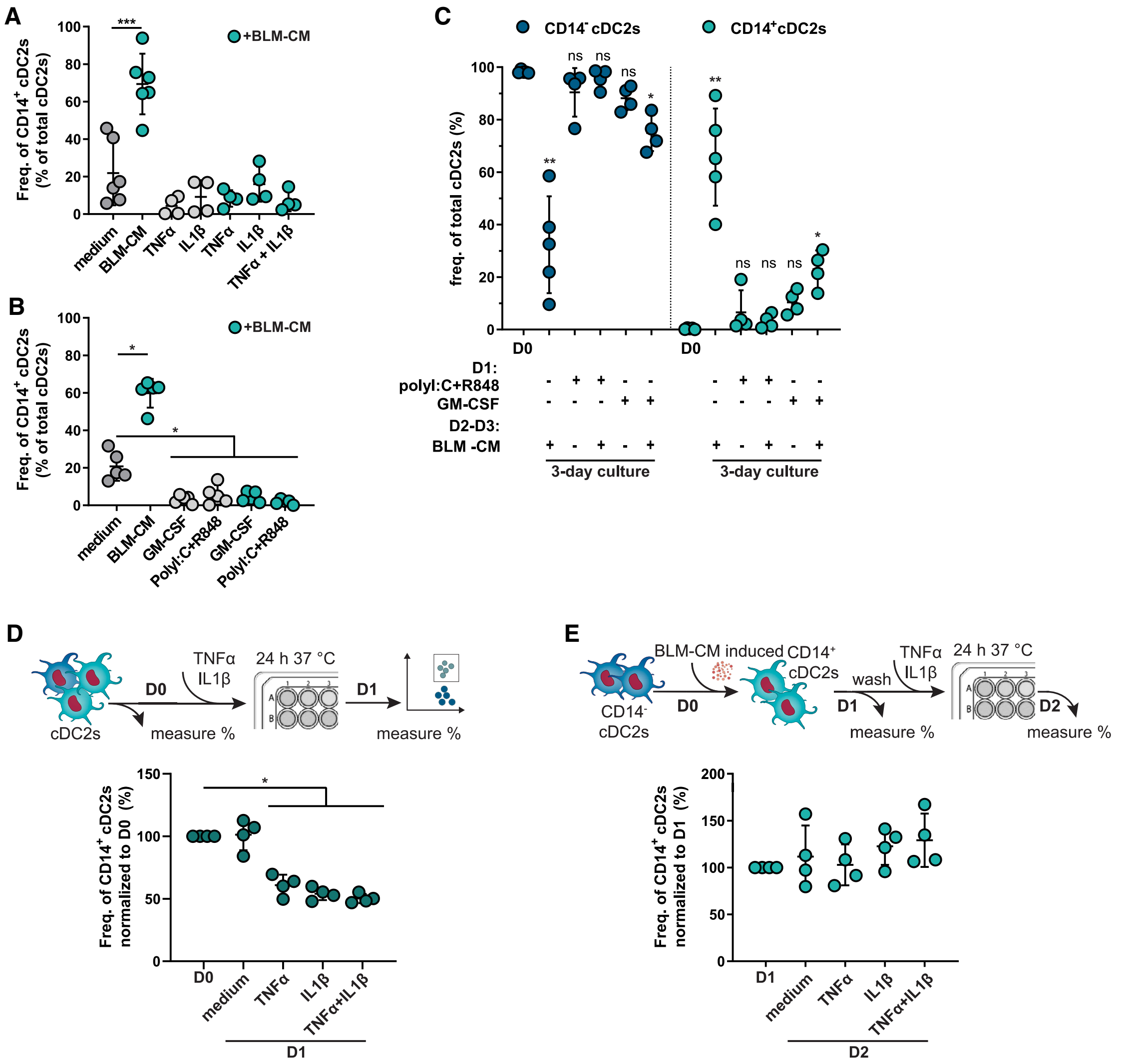

Figure 5. Healthy cDC2s have phenotypic plasticity and once matured do not convert to CD14+ cDC2s

(A and B) Frequencies of CD14+ cDC2s after culturing CD14− cDC2s 2 days in the presence or absence of 20 ng/mL TNF-α and/or IL-1β (A) or 800 U/mL GM-CSF or TLR ligands poly(I:C) (20 μg/mL) and R848 (4 μg/mL) (B), with and without BLM-CM.

(C) Frequencies of CD14− (left) and CD14+ (right) cDC2s after CD14− cDC2 isolation (D0) and after culturing with and without maturation stimuli, prior to BLM-CM.

(D and E) Schematic of the assay and graph showing percentages of CD14+ cDC2s normalized to D0 after culturing CD14+ cDC2s from HDs (D) and normalized to D1 after inducing CD14+ cDC2s with BLM-CM (E), both with 10 ng/mL TNF-α and/or IL-1β. (A–E) Each symbol represents a biological replicate (mean ± SD). Asterisks depict significance compared with D0 (A–D) or D1 (E) (RM one-way ANOVA, Dunnett's multiple comparisons test). *$p < 0.05$, **$p < 0.01$, ***$p < 0.001$. See also Figures S4D and S4E.

cDC2 transdifferentiation (Figure 6C). In addition to the induction of CD14, we assessed to what extent IL-6, M-CSF, and BLM-CM induce comparable phenotypes in CD14+ cDC2s by measuring the functional and immune regulatory markers we found for CD14+ cDC2s in NSCLC (Figure 1C). These data show that IL-6 and M-CSF combined induce a comparable phenotype to BLM-CM, including high IL-10, CD163, CD11b, CD206, and MERTK expression, while CD5 is absent, and HLA-DR reduced (Figure S5A). Direct comparison of BLM-CM-induced CD14+ cDC2s from HDs with CD14+ cDC2s isolated from NSCLC patients, both cultured for 3 days, reveals a high level of similarity as well with only HLA-DR and CD163 significantly different on BLM-CM-induced CD14+ cDC2s (Figure S5B).

Finally, to assess if the treatment improves functional characteristics of cDC2s, we analyzed allogeneic T cell proliferation induced by cDC2s after they were cultured with BLM-CM with or without drug treatment. Concordant with the phenotype data and HLA-DR expression (Figure 6E), treatment with anti-IL-6R and CSF1Ri significantly improved the ability of BLM-CM-exposed cDC2s to activate both CD4 and CD8 T cells (Figure 6D). Despite their culture in the presence of tumor-associated factors from BLM-CM, cDC2s treated with anti-IL-6R and CSF1Ri even slightly

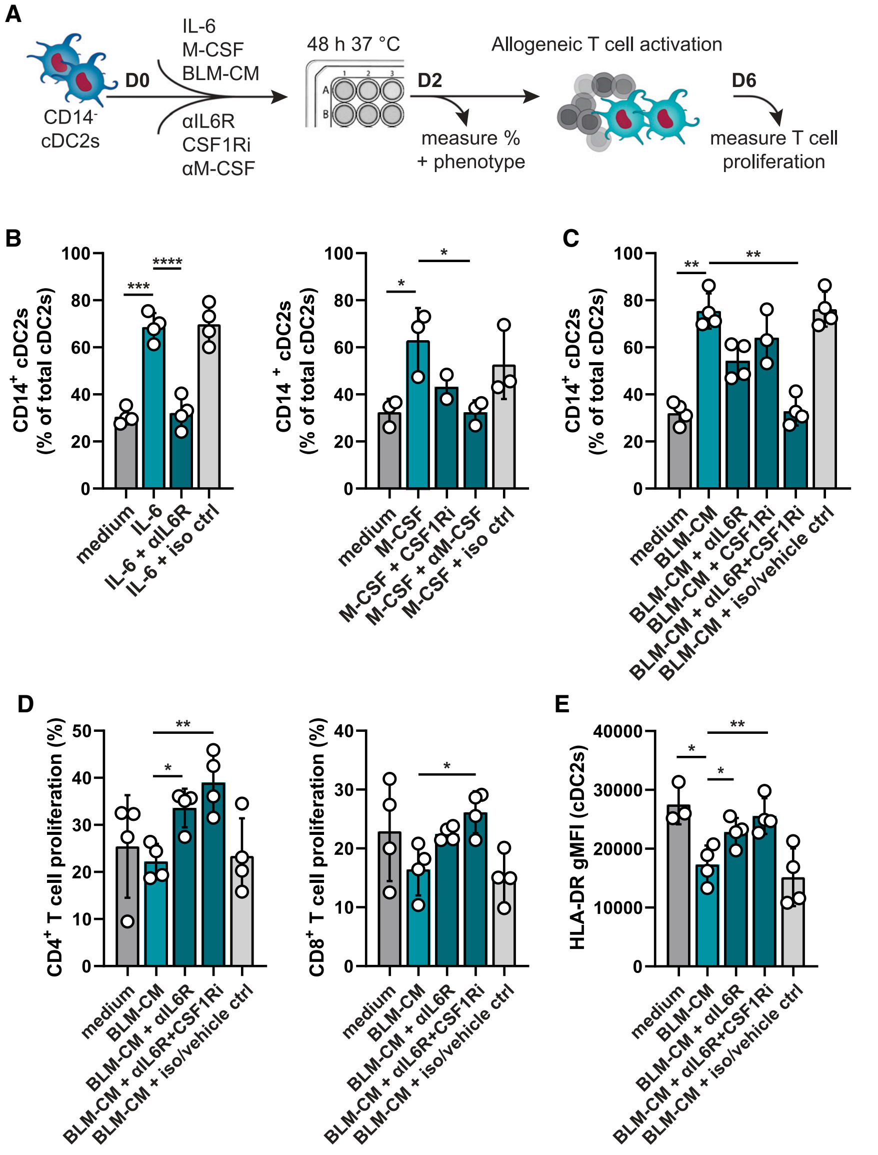

Figure 6. Modulation of cDC2s with anti-IL-6R and CSF1Ri prevents CD14+ cDC2s and enhances T cell activation

(A–C) (A) Schematic of the assays. Effect on CD14+ cDC2 frequencies after 2 days cultured with 1 ng/mL IL-6 and 10 μg/mL tocilizumab (αIL-6R) (B, left), 10 ng/mL M-CSF and 100 nM sunitinib (CSF1Ri) or 10 μg/mL αM-CSF antibody (B, right), or BLM-CM and both drugs (C).

(D) Capacity of cDC2s, cultured for 2 days with or without BLM-CM and αIL-6R + CSF1Ri to induce allogeneic CD4 and CD8 T cell proliferation after a 4-day co-culture.

(E) HLA-DR expression on cDC2s on day 2 prior to addition of T cells. (B–E) Each symbol represents a biological replicate (mean ± SD). RM one-way ANOVA, Dunnett's multiple comparisons test, vs. IL-6 (B), M-CSF (B), or the BLM-CM condition (C–E). *$p < 0.05$, **$p < 0.01$, ***$p < 0.001$, gMFI, geometric mean fluorescent intensity. See also Figure S5.

outperformed untreated medium-cultured cDC2s (Figure 6D). In summary, prevention of tumor-induced CD14+ cDC2s can be achieved with tocilizumab and sunitinib leading to an improved immunostimulatory capacity of cDC2s.

DISCUSSION

Tackling tumor-induced immune suppression is critical for improving spontaneous and immunotherapy-induced anti-tumor immune responses. In this work, we focus on tumor-induced CD14+ DCs arising in melanoma and NSCLC patients. After characterizing CD1c+CD14+ cells in NSCLC, we directed our efforts to dissecting their development driven by tumor-derived factors. Using *in vitro* assays, we provide evidence of a direct conversion of cDC2s to CD1c+CD14+CD5− DCs, with M-CSF and IL-6 as responsible factors. In addition, we provide a way to prevent CD14+ DC3 induction using anti-IL-6R antibodies and CSF1Ris, leading to improved T cell responses.

Based on our phenotypic analysis of cDC2 subsets, we describe a CD163+CD206+CD11b+HLA-DR^{low} phenotype for CD1c+CD14+ DCs in NSCLC and HDs, aligning with the DC3 phenotype. Multiple studies describing CD1c+CD14+ DCs in cancer and healthy state reported similar phenotypes, characterized by co-expression of these monocyte/macrophage markers with cDC2 markers.[4,5,8,10,12] For NSCLC, several studies report the presence of cDC2s in tumors and peripheral blood,[29–31] or tolerogenic cDC2s induced by NSCLC cells.[32] Primary NSCLC cells downregulated co-stimulatory molecules on CD1c+ DCs,[32] in accordance with our observed phenotype of CD1c+CD14+ DCs. Higher frequencies of tumor-infiltrating CD1c+ DCs coincided with significantly lower survival rates (19.2 ± 17.2 vs. 42.9 ± 15.9 months for low frequencies).[29] Aside from expanded CD163+CD14+ DCs with an anti-inflammatory phenotype reported,[31] these studies did not discriminate between CD14− and CD14+ DCs. Thus, to what extent the observed effects on patient survival and tolerogenic characteristics could be explained by the CD1c+CD14+ DC fraction remains unknown. Of note, Zilionis and co-workers use the term "DC3" in NSCLC for mature tumor-infiltrating DCs lacking CD14 expression, which do not correspond to the tumor-induced CD1c+CD14+ cells described here.[33–35] Another frequently described tumor-induced phenotype in DCs is the mregDCs (mature DCs enriched in immunostimulatory molecules). Although mregDCs show similarities with tumor-induced CD1c+CD14+ cells, such as high expression of immunoregulatory

markers, mregDCs are detected across all mature DC subsets (cDC1s, cDC2s, DC3s), thereby representing a tumor-induced cell state, while CD1c$^+$CD14$^+$ cells are cDC2 specific and present in both immature and mature states.[36] Moreover, healthy CD14$^-$ cDC2s did not convert to CD14$^+$ cDC2s once matured, suggesting that mature cDC2s lose a differentiation ability similarly to that observed by Diao et al. for murine DCs that lost their capacity to develop into macrophage-like DCs upon maturation.[37] In summary, the phenotypic hallmarks described for CD1c$^+$CD14$^+$ DCs are similar across different tumor types, to which we have added NSCLC, and resemble the recently introduced DC3s.[4–6,8,12]

In addition to DC3-related proteins (CD1c$^+$CD5$^-$CD14$^+$ CD163$^+$), the NSCLC peripheral blood CD14$^+$ cDC2s displayed higher PD-L1, MERTK, IL-10, and IDO, compared with CD14$^-$ cDC2s. High PD-L1 expression on tumor DCs dampens anti-tumor T cell responses.[13] Similarly, DCs producing IL-10 and IDO inhibit immune responses.[38] MERTK, a transmembrane receptor tyrosine kinase, is highly expressed by tolerogenic DCs and immunosuppressive macrophages and dampens the immune response.[21,22] Therefore, the here reported increased MERTK expression on CD14$^+$ cDC2s, with the highest expression detected in cancer patients, aligns with the known role of MERTK. Furthermore, the increased inhibitory proteins on CD14$^+$ cDC2s are consistent with the reduced allogeneic CD4 T cells we observed. In addition, CD14$^+$ cDC2s showed reduced tumor antigen-specific CD8 T cell activation. Considering that CD14$^+$ cDC2s are consistently found in peripheral blood of HDs where they also display reduced T cell stimulatory capacity vs. CD14$^-$ cDC2s, the tumor-induced expansion of CD14$^+$ cDC2s could be tumors exploiting a homeostatic system to hamper the anti-tumor T cell responses. Although the capability of CD14$^+$ cDC2/DC3s to induce CD4 T cell activation has been demonstrated by us and several others, some discrepancies exist. Villani et al. described DC3s as CD1c_B in healthy state and showed that they were as potent in stimulating allogeneic T cells as DC2s.[1] Although the discriminative gene set of CD1c_B cells includes CD14 and CD163 in line with our CD14$^+$ cDC2 subset phenotype, their isolation strategy included CD1c$^+$CD163$^+$ DCs from a CD14$^-$ population. The absence of CD1c$^+$CD163$^+$CD14$^+$ cells in the CD1c_B functional assays most likely contributes to the observed differences compared with our results.[1] In the study of Bourdely et al., TLR agonist-matured DC3s activated CD4 and CD8 T cells to a lower extent vs. cDC2s.[6] Further functional specializations attributed to DC3s include the differentiation of CD103$^+$CD8$^+$ T cells[6] and of IL-17$^+$CD4$^+$ T cells.[4] Taken together, variations in function across DC3 studies might relate to differences in the markers used for subset selection (CD14/CD163/CD5), DC maturation status, and tested T cell subsets. Further studies are needed to unravel the specific role of DC3s in health and in the pathophysiology of different cancers.

The enrichment of CD1c$^+$CD14$^+$ cells with low immunostimulatory capability in melanoma and NSCLC prompted us to study their development. We observed decreased CD1c$^+$CD14$^+$ DC frequencies in patients within 10 weeks after NSCLC resection, supporting the hypothesis of a tumor-driven emergence of CD1c$^+$CD14$^+$ DCs. For potential precursors, we considered cDC2s and monocytes. Monocytes because they are known for their ability to differentiate into monocyte-derived DCs. However, we demonstrate that monocytes are incapable of transdifferentiating to CD1c$^+$CD14$^+$ DCs in response to tumor cues. These findings are in accordance with the well-established distinction between the DC and monocyte lineage on developmental, phenotypic, and functional levels.[39] Although it is apparent that monocytes are not involved, the exact ontogeny of human CD14$^+$ DCs/DC3s is still a matter of debate. DC3s developing from CD34$^+$ HSPCs independent of CDPs, and therefore distinct from conventional DCs, were shown by Bourdely et al. in the presence of GM-CSF and mouse bone marrow-derived MS5 stromal cells.[6] DC3 induction from cDC2s and monocytes by GM-CSF was subsequently tested but failed to induce CD14$^+$ DCs, in agreement with our work. A second developmental pathway was found by Cytlak et al. in which DC3s and monocytes followed an IRF8^{low} pathway, while an IRF8^{high} pathway forms pDCs, cDC1s, and DC2s.[7] Besides these cDC2-independent trajectories, the pseudo-time analyses from Dutertre et al. suggested a shared lineage from cDC2s and DC3s arising from CD5$^+$ DCs that progress via CD5$^-$CD163$^-$ cells toward CD5$^-$CD163$^+$ and finally CD163$^+$CD14$^+$ cells.[4] Accordingly, we observed a direct conversion of cDC2s into CD14$^+$ cDC2s. We detected IL-6 and M-CSF, present in tumor-CM and patient serum, as inducers of CD14$^+$ cDC2s from cDC2s. Moreover, inhibiting their signaling with anti-IL-6R and CSF1Ri kept CD14$^+$ cDC2 frequencies equal to tumor-free conditions, underlining IL-6 and M-CSF as dominant drivers of tumor-induced CD14$^+$ cDC2s. These results challenge the division of DC3s as separate DC subset and support DC3s as part of the cDC2 lineage. Considering the shared developmental trajectory for cDC2s and CD14$^+$ DCs found here, tumor-induced CD14$^+$ DCs might represent a cell state induced by tumor cues since they do not fulfill the requirement of a separate progenitor as defined for DC subsets in general.[2] It would be interesting to see if DC3s can be obtained from CD34$^+$ HPSCs upon IL-6 and M-CSF exposure, as was demonstrated for GM-CSF.[6] The MS5 cells utilized in the GMDP co-cultures have been reported to produce IL-6 and M-CSF in other studies[40,41]; however, whether IL-6 and M-CSF are involved in GMDP-derived DC3s remains to be elucidated.

Our finding that simultaneous inhibition of IL-6R and CSF1R drastically reduces tumor-induced cDC2 to CD14$^+$ DC conversion creates several therapeutic possibilities. Tocilizumab and sunitinib are US FDA-approved drugs with low health risks, even when used for longer time periods.[42,43] DCs in the periphery are constantly replenished by hematopoietic progenitors. Because of this high turnover rate and limited lifespan, cDC2 modulation that prevents instead of reverting CD14$^+$ cDC2s has great potential for improving the immunostimulatory capacity of cDC2s. Of note, with tocilizumab treatment the pleiotropic effects of IL-6, which include pro- and anti-tumor effects, have to be considered. Several studies show beneficial effects of blocking IL-6 in cancer, especially when IL-6 and/or IL-6R expression are high,[44–46] indicating that selecting the cancer type is an important factor to effectively benefit from preventing IL-6-mediated pro-tumor effects, such as tumor-induced CD14$^+$ cDC2s, while limiting unwanted side effects as result of the absence of IL-6-mediated anti-tumor effects. Collectively, this warrants future investigation focused on combination therapies of sunitinib and tocilizumab

with immunotherapies aiming at T cell-mediated tumor elimination to improve cancer therapies efficacies.

In conclusion, we show that "DC3-like" CD14+ DCs can arise from cDC2s but not from monocytes in response to tumor-derived cues, which highlights that further delineation of the tumor-induced CD14+ DC and DC3 lineage is essential to precisely define the DC family. We furthermore show that IL-6 and M-CSF are strong drivers of increased abundance of immune incompetent CD14+ DC3s in the context of cancer and show that pharmacological inhibition of the IL-6R and CSF1R has potential to improve immunotherapy.

Limitations of the study

In this study, we demonstrate that CD14− cDC2s can directly convert to CD1c+CD14+CD163+ DCs driven by tumor-associated factors. When CD1c+ cells are depleted from PBMCs, CD1c+CD14+CD163+ DCs were absent after a two-day culture. These results show that the short-term tumor-induced expansion of CD1c+CD14+CD163+ DCs depends on cDC2s. However, this does not exclude that other progenitors can give rise to CD1c+CD14+CD163+ DCs over a longer period and/or sourced outside peripheral blood, which will need to be determined by future studies.

We investigated the phenotype, the ability to induce T cell proliferation in a non-autologous priming context, and the cytokine profile of CD14+ cDC2s in HDs and NSCLC patients. We supplemented these findings with tumor antigen-specific T cell assays using DC subsets from HDs, demonstrating their inferior CD8 T cell responses. Due to the limited availability of patient materials, we did not investigate CD14+ cDC2s from NSCLC patients using this experimental setup. Therefore, the capability of CD14+ cDC2s from NSCLC patients to process and present antigens remains to be addressed.

We identified IL-6 and M-CSF as dominant drivers in the CD14+ cDC2 transdifferentiation following targeted proteomics and *in vitro* validations. However, this study focused on the involvement of tumor-associated proteins without assessing the contribution of other solutes, such as lipids. These findings therefore do not exclude that other solutes can contribute to the development of tumor-induced CD14+ cDC2s.

We report that the presence of CD14+ cDC2s in melanoma patients interferes with the effectiveness of autologous DC vaccines. Our *in vitro* experiments furthermore showed that anti-IL-6R and CSF1Ri effectively prevent the development of tumor-induced CD14+ cDC2s. Further studies will be needed to assess the effectiveness of these pharmacological inhibitors *in vivo* and their potential to improve therapy efficacy when combined with DC-based cancer vaccinations.

STAR★METHODS

Detailed methods are provided in the online version of this paper and include the following:

- KEY RESOURCES TABLE
- RESOURCE AVAILABILITY
  - Lead contact
  - Materials availability
  - Data and code availability
- EXPERIMENTAL MODEL AND SUBJECT DETAILS
  - Human blood and serum samples
  - Cell culture cancer cell lines
- METHOD DETAILS
  - Isolation of human blood immune cells
  - Monocyte and cDC2 conversion experiments
  - Human cell flow cytometry
  - Allogeneic T cell proliferation assays
  - NY-ESO1 specific T cell activation assays
  - Cytokine quantification
  - PBMC cultures
  - OLINK analysis
  - RNA microarray
  - Drug treatment
- QUANTIFICATION AND STATISTICAL ANALYSIS
- ADDITIONAL RESOURCES

SUPPLEMENTAL INFORMATION

Supplemental information can be found online at https://doi.org/10.1016/j.xcrm.2023.101386.

ACKNOWLEDGMENTS

We thank healthy donors and patients for their participation. We thank Rob Woestenenk for valuable technical assistance. The Vermeulen lab is part of the Oncode Institute, which is partly funded by the Dutch Cancer Society. This research was supported by a Radboudumc PhD grant to A.M.D.B. (Nijmegen, the Netherlands) and Health Holland grants DC4Balance (LSHM18056-SGF) and ImmuneHealthSeed (LSHM22042-SGF) to I.J.M.d.V. M. Verdoes is a recipient of ERC Starting grant CHEMCHECK (679921) and a Gravity Program Institute for Chemical Immunology tenure track grant by NWO. E.H.J.G.A. received a research grant from AstraZeneca, Health Holland (LSHM18090) and Dutch Cancer Society (KWF-12493).

AUTHOR CONTRIBUTIONS

Conceptualization, A.M.D.B., M. Verdoes, and I.J.M.d.V.; investigation, A.M.D.B., A.H.D., G.F.-G., and G.B.; data analysis, A.M.D.B., R.J.R., and S.S.; resources – DONAN patient samples, E.H.J.G.A. and B.P.; writing – original draft, A.M.D.B., writing – review & editing, A.M.D.B., M. Verdoes, and I.J.M.d.V.; visualization, A.M.D.B., M. Verdoes, and I.J.M.d.V.; supervision, M. Verdoes, I.J.M.d.V., and M. Vermeulen of S.S.; funding acquisition, A.M.D.B., E.H.J.G.A., M. Verdoes, and I.J.M.d.V.

DECLARATION OF INTERESTS

The authors declare no competing interests.

Received: December 8, 2022
Revised: September 29, 2023
Accepted: December 20, 2023
Published: January 18, 2024

REFERENCES

1. Villani, A.C., Satija, R., Reynolds, G., Sarkizova, S., Shekhar, K., Fletcher, J., Griesbeck, M., Butler, A., Zheng, S., Lazo, S., et al. (2017). Single-cell RNA-seq reveals new types of human blood dendritic cells, monocytes, and progenitors. Science *356*, eaah4573.
2. Ginhoux, F., Guilliams, M., and Merad, M. (2022). Expanding dendritic cell nomenclature in the single-cell era. Nat. Rev. Immunol. *22*, 67–68.

3. Guilliams, M., Ginhoux, F., Jakubzick, C., Naik, S.H., Onai, N., Schraml, B.U., Segura, E., Tussiwand, R., and Yona, S. (2014). Dendritic cells, monocytes and macrophages: a unified nomenclature based on ontogeny. Nat. Rev. Immunol. *14*, 571–578.

4. Dutertre, C.A., Becht, E., Irac, S.E., Khalilnezhad, A., Narang, V., Khalilnezhad, S., Ng, P.Y., van den Hoogen, L.L., Leong, J.Y., Lee, B., et al. (2019). Single-Cell Analysis of Human Mononuclear Phagocytes Reveals Subset-Defining Markers and Identifies Circulating Inflammatory Dendritic Cells. Immunity *51*, 573–589.e8.

5. Kvedaraite, E., and Ginhoux, F. (2022). Human dendritic cells in cancer. Sci. Immunol. *7*, eabm9409.

6. Bourdely, P., Anselmi, G., Vaivode, K., Ramos, R.N., Missolo-Koussou, Y., Hidalgo, S., Tosselo, J., Nuñez, N., Richer, W., Vincent-Salomon, A., et al. (2020). Transcriptional and Functional Analysis of CD1c(+) Human Dendritic Cells Identifies a CD163(+) Subset Priming CD8(+)CD103(+) T Cells. Immunity *53*, 335–352.e8.

7. Cytlak, U., Resteu, A., Pagan, S., Green, K., Milne, P., Maisuria, S., McDonald, D., Hulme, G., Filby, A., Carpenter, B., et al. (2020). Differential IRF8 Transcription Factor Requirement Defines Two Pathways of Dendritic Cell Development in Humans. Immunity *53*, 353–370.e8.

8. Bakdash, G., Buschow, S.I., Gorris, M.A.J., Halilovic, A., Hato, S.V., Sköld, A.E., Schreibelt, G., Sittig, S.P., Torensma, R., Duiveman-de Boer, T., et al. (2016). Expansion of a BDCA1+CD14+ Myeloid Cell Population in Melanoma Patients May Attenuate the Efficacy of Dendritic Cell Vaccines. Cancer Res. *76*, 4332–4346.

9. Binnewies, M., Mujal, A.M., Pollack, J.L., Combes, A.J., Hardison, E.A., Barry, K.C., Tsui, J., Ruhland, M.K., Kersten, K., Abushawish, M.A., et al. (2019). Unleashing Type-2 Dendritic Cells to Drive Protective Antitumor CD4+ T Cell Immunity. Cell *177*, 556–571.e16.

10. Michea, P., Noël, F., Zakine, E., Czerwinska, U., Sirven, P., Abouzid, O., Goudot, C., Scholer-Dahirel, A., Vincent-Salomon, A., Reyal, F., et al. (2018). Adjustment of dendritic cells to the breast-cancer microenvironment is subset specific. Nat. Immunol. *19*, 885–897.

11. Segura, E., Touzot, M., Bohineust, A., Cappuccio, A., Chiocchia, G., Hosmalin, A., Dalod, M., Soumelis, V., and Amigorena, S. (2013). Human Inflammatory Dendritic Cells Induce Th17 Cell Differentiation. Immunity *38*, 336–348.

12. Di Blasio, S., van Wigcheren, G.F., Becker, A., van Duffelen, A., Gorris, M., Verrijp, K., Stefanini, I., Bakker, G.J., Bloemendal, M., Halilovic, A., et al. (2020). The tumour microenvironment shapes dendritic cell plasticity in a human organotypic melanoma culture. Nat. Commun. *11*, 2749.

13. Peng, Q., Qiu, X., Zhang, Z., Zhang, S., Zhang, Y., Liang, Y., Guo, J., Peng, H., Chen, M., Fu, Y.-X., and Tang, H. (2020). PD-L1 on dendritic cells attenuates T cell activation and regulates response to immune checkpoint blockade. Nat. Commun. *11*, 4835.

14. Wculek, S.K., Cueto, F.J., Mujal, A.M., Melero, I., Krummel, M.F., and Sancho, D. (2020). Dendritic cells in cancer immunology and immunotherapy. Nat. Rev. Immunol. *20*, 7–24.

15. Scarlett, U.K., Rutkowski, M.R., Rauwerdink, A.M., Fields, J., Escovar-Fadul, X., Baird, J., Cubillos-Ruiz, J.R., Jacobs, A.C., Gonzalez, J.L., Weaver, J., et al. (2012). Ovarian cancer progression is controlled by phenotypic changes in dendritic cells. J. Exp. Med. *209*, 495–506.

16. Berghmans, T., Dingemans, A.-M., Hendriks, L.E.L., and Cadranel, J. (2020). Immunotherapy for nonsmall cell lung cancer: a new therapeutic algorithm. Eur. Respir. J. *55*, 1901907.

17. Yarchoan, M., Hopkins, A., and Jaffee, E.M. (2017). Tumor Mutational Burden and Response Rate to PD-1 Inhibition. N. Engl. J. Med. *377*, 2500–2501.

18. Alexandrov, L.B., Nik-Zainal, S., Wedge, D.C., Aparicio, S.A.J.R., Behjati, S., Biankin, A.V., Bignell, G.R., Bolli, N., Borg, A., Børresen-Dale, A.L., et al. (2013). Signatures of mutational processes in human cancer. Nature *500*, 415–421.

19. Sambi, M., Bagheri, L., and Szewczuk, M.R. (2019). Current Challenges in Cancer Immunotherapy: Multimodal Approaches to Improve Efficacy and Patient Response Rates. JAMA Oncol. *2019*, 4508794.

20. Schreibelt, G., Bol, K.F., Westdorp, H., Wimmers, F., Aarntzen, E.H.J.G., Duiveman-de Boer, T., van de Rakt, M.W.M.M., Scharenborg, N.M., de Boer, A.J., Pots, J.M., et al. (2016). Effective Clinical Responses in Metastatic Melanoma Patients after Vaccination with Primary Myeloid Dendritic Cells. Clin. Cancer Res. *22*, 2155–2166.

21. Giroud, P., Renaudineau, S., Gudefin, L., Calcei, A., Menguy, T., Rozan, C., Mizrahi, J., Caux, C., Duong, V., and Valladeau-Guilemond, J. (2020). Expression of TAM-R in Human Immune Cells and Unique Regulatory Function of MerTK in IL-10 Production by Tolerogenic DC. Front. Immunol. *11*, 564133.

22. Cabezón, R., Carrera-Silva, E.A., Flórez-Grau, G., Errasti, A.E., Calderón-Gómez, E., Lozano, J.J., España, C., Ricart, E., Panés, J., Rothlin, C.V., and Benítez-Ribas, D. (2015). MERTK as negative regulator of human T cell activation. J. Leukoc. Biol. *97*, 751–760.

23. Schrøder, M., Melum, G.R., Landsverk, O.J.B., Bujko, A., Yaqub, S., Gran, E., Aamodt, H., Bækkevold, E.S., Jahnsen, F.L., and Richter, L. (2016). CD1c-Expression by Monocytes - Implications for the Use of Commercial CD1c+ Dendritic Cell Isolation Kits. PLoS One *11*, e0157387.

24. Borriello, F., Iannone, R., Di Somma, S., Vastolo, V., Petrosino, G., Visconte, F., Raia, M., Scalia, G., Loffredo, S., Varricchi, G., et al. (2017). Lipopolysaccharide-Elicited TSLPR Expression Enriches a Functionally Discrete Subset of Human CD14(+) CD1c(+) Monocytes. J. Immunol. *198*, 3426–3435.

25. da Cunha, B.R., Domingos, C., Stefanini, A.C.B., Henrique, T., Polachini, G.M., Castelo-Branco, P., and Tajara, E.H. (2019). Cellular Interactions in the Tumor Microenvironment: The Role of Secretome. J. Cancer *10*, 4574–4587.

26. Wu, X., Xu, F., Liu, J., and Wang, G. (2017). Comparative study of dendritic cells matured by using IL-1β, IL-6, TNF-α and prostaglandins E2 for different time span. Exp. Ther. Med. *14*, 1389–1394.

27. Sallusto, F., and Lanzavecchia, A. (1994). Efficient presentation of soluble antigen by cultured human dendritic cells is maintained by granulocyte/macrophage colony-stimulating factor plus interleukin 4 and downregulated by tumor necrosis factor alpha. J. Exp. Med. *179*, 1109–1118.

28. Lejeune, F.J., Liénard, D., Matter, M., and Rüegg, C. (2006). Efficiency of recombinant human TNF in human cancer therapy. Cancer Immun. *6*, 6.

29. Tabarkiewicz, J., Rybojad, P., Jablonka, A., and Rolinski, J. (2008). CD1c+ and CD303+ dendritic cells in peripheral blood, lymph nodes and tumor tissue of patients with non-small cell lung cancer. Oncol. Rep. *19*, 237–243.

30. Stankovic, B., Bjørhovde, H.A.K., Skarshaug, R., Aamodt, H., Frafjord, A., Müller, E., Hammarström, C., Beraki, K., Bækkevold, E.S., Woldbæk, P.R., et al. (2018). Immune Cell Composition in Human Non-small Cell Lung Cancer. Front. Immunol. *9*, 3101.

31. Kim, N., Kim, H.K., Lee, K., Hong, Y., Cho, J.H., Choi, J.W., Lee, J.-I., Suh, Y.-L., Ku, B.M., Eum, H.H., et al. (2020). Single-cell RNA sequencing demonstrates the molecular and cellular reprogramming of metastatic lung adenocarcinoma. Nat. Commun. *11*, 2285.

32. Lu, Y., Xu, W., Gu, Y., Chang, X., Wei, G., Rong, Z., Qin, L., Chen, X., and Zhou, F. (2019). Non-small Cell Lung Cancer Cells Modulate the Development of Human CD1c(+) Conventional Dendritic Cell Subsets Mediated by CD103 and CD205. Front. Immunol. *10*, 2829.

33. Gerhard, G.M., Bill, R., Messemaker, M., Klein, A.M., and Pittet, M.J. (2021). Tumor-infiltrating dendritic cell states are conserved across solid human cancers. J. Exp. Med. *218*, e20200264.

34. Zilionis, R., Engblom, C., Pfirschke, C., Savova, V., Zemmour, D., Saatcioglu, H.D., Krishnan, I., Maroni, G., Meyerovitz, C.V., Kerwin, C.M., et al. (2019). Single-Cell Transcriptomics of Human and Mouse Lung Cancers Reveals Conserved Myeloid Populations across Individuals and Species. Immunity *50*, 1317–1334.e10.

CellPress
OPEN ACCESS

35. Segura, E. (2022). Human dendritic cell subsets: An updated view of their ontogeny and functional specialization. Eur. J. Immunol. *52*, 1759–1767.

36. Del Prete, A., Salvi, V., Soriani, A., Laffranchi, M., Sozio, F., Bosisio, D., and Sozzani, S. (2023). Dendritic cell subsets in cancer immunity and tumor antigen sensing. Cell. Mol. Immunol. *20*, 432–447.

37. Diao, J., Mikhailova, A., Tang, M., Gu, H., Zhao, J., and Cattral, M.S. (2012). Immunostimulatory conventional dendritic cells evolve into regulatory macrophage-like cells. Blood *119*, 4919–4927.

38. DeVito, N.C., Plebanek, M.P., Theivanthiran, B., and Hanks, B.A. (2019). Role of Tumor-Mediated Dendritic Cell Tolerization in Immune Evasion. Front. Immunol. *10*, 2876.

39. Anderson, D.A., Dutertre, C.-A., Ginhoux, F., and Murphy, K.M. (2021). Genetic models of human and mouse dendritic cell development and function. Nat. Rev. Immunol. *21*, 101–115.

40. Heberlein, C., Friel, J., Itoh, K., Medlock, E., Li, L., Nakayama, N., Stocking, C., Geldmacher, M., and Ostertag, W. (2006). Involvement of CSF-1 in generating a stroma-independent hematopoietic stem cell line. J. Cell. Physiol. *206*, 556–562.

41. Nakayama, A., Matsui, H., Fukushima, T., Ichikawa, H., Yamada, K., Amao, T., Hosono, M., and Sugimoto, K. (2006). Murine serum obtained from bone marrow-transplanted mice promotes the proliferation of hematopoietic stem cells by co-culture with MS-5 murine stromal cells. Growth Factors *24*, 55–65.

42. Jones, G., and Panova, E. (2018). New insights and long-term safety of tocilizumab in rheumatoid arthritis. Ther. Adv. Musculoskelet. Dis. *10*, 195–199.

43. Porta, C., Gore, M.E., Rini, B.I., Escudier, B., Hariharan, S., Charles, L.P., Yang, L., DeAnnuntis, L., and Motzer, R.J. (2016). Long-term Safety of Sunitinib in Metastatic Renal Cell Carcinoma. Eur. Urol. *69*, 345–351.

44. Hong, C., Schubert, M., Tijhuis, A.E., Requesens, M., Roorda, M., van den Brink, A., Ruiz, L.A., Bakker, P.L., van der Sluis, T., Pieters, W., et al. (2022). cGAS–STING drives the IL-6-dependent survival of chromosomally instable cancers. Nature *607*, 366–373.

45. Bent, E.H., Millán-Barea, L.R., Zhuang, I., Goulet, D.R., Fröse, J., and Hemann, M.T. (2021). Microenvironmental IL-6 inhibits anti-cancer immune responses generated by cytotoxic chemotherapy. Nat. Commun. *12*, 6218.

46. Huseni, M.A., Wang, L., Klementowicz, J.E., Yuen, K., Breart, B., Orr, C., Liu, L.F., Li, Y., Gupta, V., Li, C., et al. (2023). CD8(+) T cell-intrinsic IL-6 signaling promotes resistance to anti-PD-L1 immunotherapy. Cell Rep. Med. *4*, 100878.

47. Wickham, H., Averick, M., Bryan, J., Chang, W., McGowan, L., François, R., Grolemund, G., Hayes, A., Henry, L., Hester, J., et al. (2019). Welcome to the Tidyverse. J. Open Source Softw. *4*, 1686.

48. Schauberger P, W.A. (2022). Openxlsx: Read, Write and Edit Xlsx Files. https://ycphs.github.io/openxlsx/index.html. https://github.com/ycphs/openxlsx.

49. R, K. (2019). _pheatmap: Pretty Heatmaps_. R Package version 1.0.12. https://CRAN.R-project.org/package=pheatmap.

50. Blighe, K.R.S., and Lewis, M. (2022). _EnhancedVolcano: Publication-Ready Volcano Plots with Enhanced Colouring and Labeling_. R Package Version 1.14.0. https://github.com/kevinblighe/EnhancedVolcano.

51. Ritchie, M.E., Phipson, B., Wu, D., Hu, Y., Law, C.W., Shi, W., and Smyth, G.K. (2015). limma powers differential expression analyses for RNA-sequencing and microarray studies. Nucleic Acids Res. *43*, e47.

52. A. Liaw, o.R.G., M. Maechler, W. Huber, G. Warnes heatmap.2, Levi Waldron heatmap.3.

STAR★METHODS

KEY RESOURCES TABLE

REAGENT or RESOURCE	SOURCE	IDENTIFIER
Antibodies		
BV785 anti-human CD45 antibody, clone HI30	Biolegend	(BioLegend Cat# 304048, RRID:AB_2563129)
APC cy7 anti-human CD11b antibody, clone ICRF44	Biolegend	(BioLegend Cat# 301342, RRID:AB_2563395)
APC anti-human CD11c antibody, clone B-ly6	BD Biosciences	(BD Biosciences Cat# 559877, RRID:AB_398680)
APC anti-human CD14 antibody, clone M5E2	Biolegend	(BioLegend Cat# 301808, RRID:AB_314190)
APC-H7 anti-human CD14 antibody, clone MφP9	BD Biosciences	(BD Biosciences Cat# 560180, RRID:AB_1645464)
PE anti-human CD163 antibody, clone GHI/61	BD Biosciences	(BD Biosciences Cat# 556018, RRID:AB_396296)
PerCP anti-human CD19 antibody, clone 4G7	BD Biosciences	(BD Biosciences Cat# 345778, RRID:AB_2868806)
BV421 anti-human CD1c antibody, clone L161	Biolegend	(BioLegend Cat# 331526, RRID:AB_10962909)
PE anti-human CD1c antibody, clone AD5-8E7	Miltenyi	(Miltenyi Biotec Cat# 130-113-302, RRID:AB_2726081)
FITC anti-human CD20 antibody, clone L27	BD Biosciences	(BD Biosciences Cat# 345792, RRID:AB_2868818)
PE anti-human CD206 antibody, clone 19.2(RUO)	BD Biosciences	(BD Biosciences Cat# 555954, RRID:AB_396250)
FITC anti-human CD5 antibody, clone L17f12	eBioscience	(Thermo Fisher Scientific Cat# 11-0058-42, RRID:AB_1944383)
PerCP anti-human HLA-DR antibody, clone L243	Biolegend	(BioLegend Cat# 307628, RRID:AB_893566)
BV510 anti-human HLA-DR antibody, clone L243	Biolegend	(BioLegend Cat# 307646, RRID:AB_2561948)
FITC anti-human HLA-ABC antibody, clone REA230	Miltenyi	(Miltenyi Biotec Cat# 130-101-446, RRID:AB_2652080)
PE-Cy7 anti-human CD80 antibody, clone L307.4	BD Biosciences	(BD Biosciences Cat# 561135, RRID:AB_10561688)
PerCP-eFluor710 anti-human CD80 antibody, clone 2D10.4	eBioscience	(Thermo Fisher Scientific Cat# 46-0809-42, RRID:AB_10548359)
PE anti-human CD86 antibody, clone FUN-1	BD Biosciences	(BD Biosciences Cat# 555658, RRID:AB_396013)
APC anti-human CD86 antibody, clone FUN-1	BD Biosciences	(BD Biosciences Cat# 555660, RRID:AB_398608)
AF488 anti-human IDO1 antibody, clone #700838	R&D systems	(R and D Systems Cat# IC6030G, RRID:AB_10997134)
PE-Cy7 anti-human IL-10 antibody, clone JES3-9D7	Biolegend	(BioLegend Cat# 501420, RRID:AB_2125385)
PE-Cy7 anti-human Mertk antibody, clone 590H11G1E3	Biolegend	(BioLegend Cat# 367609, RRID:AB_2687286)
PE-Cy7 anti-human PD-L1 antibody, clone MIH1	BD Biosciences	(BD Biosciences Cat# 558017, RRID:AB_396986)
FITC anti-human CD3 antibody, clone HIT3a	BD Biosciences	(BD Biosciences Cat# 555339, RRID:AB_395745)
FITC anti-human CD56 antibody, clone NCAM16.2	BD Biosciences	(BD Biosciences Cat# 345811, RRID:AB_2868832)
BV421 anti-human CD4 antibody, clone RPA-T4	BD Biosciences	(BD Biosciences Cat# 562424, RRID:AB_11154417)
APC anti-human CD8 antibody, clone RPA-T8	BD Biosciences	(BD Biosciences Cat# 555369, RRID:AB_398595)
PE-Cy7 anti-human CD25 antibody, clone BC96	Biolegend	(BioLegend Cat# 302612, RRID:AB_314282)
BV510 anti-human CD3 antibody, clone SK7	Biolegend	(BioLegend Cat# 344828, RRID:AB_2563704)
PE anti-human CD3 antibody, clone HIT3a	BD Biosciences	(BD Biosciences Cat# 555340, RRID:AB_395746)
PE anti-human CD56 antibody, clone 5.1H11	Biolegend	(BioLegend Cat# 981202, RRID:AB_2715758)
PE anti-human CD20 antibody, clone 2H7	Biolegend	(BioLegend Cat# 302306, RRID:AB_314254)
BV605 anti-human CD8 antibody, clone G42-8	BD BioSciences	(BD Biosciences Cat# 743066, RRID:AB_2741260)
PE anti-human PD-1 antibody, clone MIH4	BD BioSciences	(BD Biosciences Cat# 557946, RRID:AB_647199)
BV786 anti-human CD4 antibody, clone SK3	BD BioSciences	(BD Biosciences Cat# 563877, RRID:AB_2738462)
FITC anti-human CD127 antibody, clone A019D5	Biolegend	(BioLegend Cat# 351312, RRID:AB_10897643)
BV605 anti-human CD163 antibody, clone GHI/61	Biolegend	(BioLegend Cat# 333616, RRID:AB_2616879)

(*Continued on next page*)

Continued

REAGENT or RESOURCE	SOURCE	IDENTIFIER
FITC anti-human CD206 antibody, clone 19.2	BD BioSciences	(BD Biosciences Cat# 551135, RRID:AB_394065)
PE anti-human CD11b, clone ICRF44	Biolegend	(BioLegend Cat# 301306, RRID:AB_314158)
PE anti-human CD80, clone L307.4	BD BioSciences	(BD Biosciences Cat# 557227, RRID:AB_396606)
PerCPCy5.5 anti-human CD16, clone 3G8	BD BioSciences	(BD Biosciences Cat# 560717, RRID:AB_1727434)
BV785 anti-human CD11c, clone 3.9	Biolegend	(BioLegend Cat# 301644, RRID:AB_2565779)
FITC anti-human CD34, clone 561	Biolegend	(BioLegend Cat# 343604, RRID:AB_1732005)
Biological samples		
PBMCs from healthy donors (Buffycoat)	Sanquin, Nijmegen, The Netherlands	N/A
PBMCs from melanoma patients	Department of Tumor Immunology, Radboudumc, Nijmegen, The Netherlands	N/A
PBMCs from NSCLC patients	Department of Pulmonology, Radboudumc, Nijmegen, The Netherlands	N/A
Serum from melanoma patients	Department of Tumor Immunology, Radboudumc, Nijmegen, The Netherlands	N/A
Serum from healthy donors	Department of Tumor Immunology, Radboudumc, Nijmegen, The Netherlands	N/A
Chemicals, peptides, and recombinant proteins		
E780 Fixable viability dye (1 in 1000)	ThermoFisher Scientific	65-0865-14
E506 Fixable viability dye (1 in 1000)	ThermoFisher Scientific	65-0866-14
CellTrace^{TM} CFSE (final conc 2.5μM)	ThermoFisher Scientific	C34554
CellTrace^{TM} Violet (final conc 2.5μM)	ThermoFisher Scientific	C34557
TNFalpha	Miltenyi	130-094-014
IL1beta	Miltenyi	130-093-898
IL-6	Miltenyi	130-093-933
GM-CSF	Miltenyi	130-093-868
CD40	Biolegend	777202
CXCL5	Biolegend	573406
MMP7	Biolegend	761302
PD-L1	Biolegend	762504
TNFRSF12A	Biolegend	769104
CXCL1	Biolegend	574402
M-CSF	PeproTech	300–25
Tocilizumab (RoActemra)	Roche	EU/1/08/492
Sunitinib (Sutent)	Pfizer	EU/1/06/347/006
Ultra-LEAF Purified anti-human M-CSF antibody (Clone A16067H)	Biolegend	699203
Ultra-LEAF Purified Human IgG1 Isotype control (Clone QA16A12)	Biolegend	403502
mouse IgG2b Isotype control	R&D systems	MAB004
Stattic	BioTechne	Cat. no. 2789
Tofacitinib (CP-690550)	Selleckchem	Cat. no. S2789
PepTivator NY-ESO-1	Miltenyi Biotec	170-076-137
NY-ESO-I$_{p157-165}$ peptide (SLLMWITQC)	GenScript	Custom synthesized
NY-ESO-I$_{p117-143}$ peptide (PVPGVLLKEFTVSGNILTIRLTAADHR)	GenScript	Custom synthesized

(*Continued on next page*)

Continued		
REAGENT or RESOURCE	SOURCE	IDENTIFIER
$OVA_{257\text{-}164}$ peptide (SIINFEKL) (irrelevant peptide)	Invivogen	Catalog code: vac-sin
Critical commercial assays		
LEGENDplex™ Human CD8/NK Panel (13-plex)	Biolegend	Cat. no. 741065
LEGENDplex™ Human Inflammation Panel 1 (13-plex)	Biolegend	Cat. no. 740809
Deposited data		
RNA microarray data raw reads and processed data	This paper	GEO: GSE218218
Olink Target 96 Immuno-Oncology panel	This paper	Table S5
Experimental models: Cell lines		
BLM	AIMM Therapeutics	(RRID:CVCL_7035)
Mel-624	ATCC	(RRID:CVCL_8054)
A375	ATCC	(RRID:CVCL_0132)
FM3	NKI	(RRID:CVCL_2046)
MeWo	NKI	(RRID:CVCL_0445)
Mel603	LUMC	(RRID:CVCL_RA52)
A549	ATCC	(RRID:CVCL_0023)
Oligonucleotides		
RNA encoding NY-ESO-1 TCRα+β for HLA-A*02:01	BioNTech	Custom synthesized
RNA encoding NY-ESO-1 TCRα+β for HLA-DRB1*04:01	BioNTech	Custom synthesized
Software and algorithms		
FlowJo V10	BD	https://www.flowjo.com
GraphPad Prism software (V8)	GraphPad Software	https://www.graphpad.com
R4.2.1	The R foundation	https://www.r-project.org

RESOURCE AVAILABILITY

Lead contact

Additional information and requests for resources and reagents should be directed to and will be fulfilled by the lead contact, I. Jolanda M. de Vries (Jolanda.deVries@radboudumc.nl).

Materials availability

This study did not generate new unique reagents.

Data and code availability

(1) RNA micro-array dataset is deposited in the Genome Expression Omnibus (GSE218218). Accession number is listed in the key resources table. Olink Target 96 Immuno-Oncology panel data is provided in the supplementary information (Table S5).
(2) This paper does not report original code, all used R packages are reported in the STAR Methods under Quantification and Statistical Analysis.
(3) Any additional information required to reanalyze the data reported in this paper is available from the lead contact upon request.

EXPERIMENTAL MODEL AND SUBJECT DETAILS

Human blood and serum samples

Human peripheral blood mononuclear cells (PBMCs) were obtained from buffy coats from healthy volunteers that provided informed consent (Sanquin). PBMCs from NSCLC patients for functional assays were obtained from apheresis material from participants of the DONAN trial (NCT03853187). For flow cytometry analysis of cDC2 frequencies over time, PBMCs were obtained from blood tubes from NSCLC patients (DONAN trial) at baseline, prior to scheduled resection of NSCLC after two courses of

Durvalumab (MEDI4736) at a fixed dose of 750 mg Q2W intravenously, and lastly during follow-up three months after baseline date. Serum samples were obtained from four stage IV melanoma patients (study KUN1997-0042) and six healthy subjects. All studies from which human material is included are conducted according to the principles of the Declaration of Helsinki and adhere to the Dutch Medical Research Involving Human Subjects Act (WMO) and Good Clinical Practice guidelines. Approval was granted by the Medical Ethical Committee Arnhem-Nijmegen (CMO). Written informed consent was obtained from all participants. Information regarding age, gender, and disease status of patients included in this work is listed in Table S1 for melanoma patients and Table S2 for NSCLC patients.

Cell culture cancer cell lines

Human melanoma cells MeWo and FM3 were kindly provided by the Netherlands Cancer Institute (NKI), Mel603 by Leiden University Medical Center (LUMC). BLM cells were obtained from AIMM Therapeutics (Amsterdam, the Netherlands). Human melanoma cells A375, Mel-624, and the human lung epithelial carcinoma cell line A549 were authenticated by American Type Culture Collection (ATCC). All cell lines were tested mycoplasma free. Cell lines were maintained by culture in Dulbecco's Modified Eagle Medium (DMEM, GIBCO) supplemented with 10% heat inactivated fetal bovine serum (FBS) and Antibiotic-Antimycotic (Thermofisher) in humidified incubators at 37°C and 5% CO_2. To obtain tumor-conditioned medium (CM), supernatant was collected after a four-day culture period or, when indicated, after a 24 h culture in serum-free medium. Conditioned medium was centrifuged at 1500 rpm for 5 min to pellet remaining cells and cell debris, aliquoted and stored at −20°C until further use.

METHOD DETAILS

Isolation of human blood immune cells

PBMCs were isolated using Lymphoprep (Axis-Shield PoC AS). $CD14^+$-monocytes were isolated directly from PBMCs using CD14 MACS microbeads combined with FcR Blocking Reagent (Miltenyi). Prior to isolation of $CD1c^+CD14^-$ cells, PBMCs were depleted for CD19 and CD14 using MACS microbeads and LD columns. Positive selection of CD1c was subsequently performed using the CD1c(BDCA1) DC isolation kit (Miltenyi) following manufacturer's protocol. Purity was assessed by staining with CD1c-BV421, CD14-APC-H7, CD20-FITC, CD3-PE and acquisition on BD FACSVerse or BD FACSLyric (Figure S3B). To obtain separate $CD1c^+CD14^-$ and $CD1c^+CD14^+$ populations, and $CD14^+$-monocytes for RNA analysis, PBMCs treated with FcR blocker and depleted for CD3 (CD3 was omitted when autologous T-cells were required), CD19, and CD56 using MACS microbeads, followed by CD1c positive selection, were stained with the appropriate sorting panel for 30 min at 4°C in sterile polypropylene round-bottom tubes (Falcon) (Table S4). Sorting was performed on a BD FACSAria or BD FACSMelody sorter (gating strategy in Figures S6A and S6B). Allogeneic T-cells for T cell activation assays were isolated using a Pan T-cells Isolation Kit (Miltenyi), according to the manufacturer's protocol. $CD8^+$ and $CD4^+$ T-cells were isolated from the unlabeled fraction after CD1c isolation using untouched $CD8^+$ T cell Isolation Kit (Miltenyi, 130-096-495) and untouched $CD4^+$ T cell Isolation Kit (Miltenyi, 130-096-533) from HLA-A*02:01$^+$ or HLA-DRB1*04:01$^+$ donors, respectively, according to the manufacturer's instructions.

Cultures with primary cDC2s, monocytes, and T-cells, were performed in X-VIVO 15 (Lonza) supplemented with 2% human serum (HS) (Sigma-Aldrich).

Monocyte and cDC2 conversion experiments

For all cell culture conversion experiments, between 50 000–100 000 cDC2s or monocytes were cultured in 200 μL X-VIVO 2% HS in round bottom 96-well plate for indicated time periods. Co-cultures with BLM and A549 cells were performed in 1:1 ratio and monocultures with 40% serum or 50% CM.

For phenotypic plasticity of cDC2s, cells were incubated with the indicated concentrations of TNFα, IL1β, GM-CSF (all Miltenyi), or 20 μg/mL poly I:C (Invivogen) and 4 μg/mL R848 (Invivogen), with and without 50% BLM-CM for the marked time periods.

To assess the effect of human recombinant factors on cDC2s, recombinant cytokines (all carrier-free) were used in the following final concentrations: 20 ng/mL TNFα, 20 ng/mL IL1β, 1 ng/mL IL-6, 20 ng/mL GM-CSF, (all Miltenyi), 2 ng/mL CD40, 2.5 ng/mL CXCL5, 2 ng/mL MMP7, 2 ng/mL PD-L1, 1 ng/mL TNFRSF12A, 2 ng/mL CXCL1 (all Biolegend) or 10 ng/mL M-CSF (PeproTech).

Human cell flow cytometry

Antibodies used for fluorescence-activated cells sorting (FACS) and flow cytometry are all listed in the key resource table with corresponding dilutions in Table S6 and used panels in Table S4. Gating strategies are shown in Figure S6. Briefly, flow cytometry stainings were performed in V-bottom 96 well plates (Greiner Bio-one) at 4°C protected from light. Cells were washed and incubated with eBioscience Fixable Viability Dye e506 or e780 (Thermo) for 30 min in PBS, washed, and incubated for 15 min with FcR Blocking Reagent (Miltenyi). Next, cells were stained with directly labeled primary antibodies for 30 min. For intra-cellular stainings, cells were fixed and permeabilized with BD Cytofix/Cytoperm (BD Biosciences), according to the manufacturer's instructions. Anti-mouse Ig, κ/Negatieve Control Compensation Particles Set (BD) was used for single stain controls. Flow cytometry acquisition was performed on a BD FACSVerse or BD FACSLyric and FlowJo v.10 (Tree Star) was used to analyze the data.

Allogeneic T cell proliferation assays

Allogeneic Pan T-cells from HDs were labeled with 2.5 μM CellTrace CFSE dye (ThermoFisher) for 10 min at 37°C. A total of 6 000 cells from drug-treated cDC2s or sorted cDC2 subsets were co-cultured with 30 000 CFSE-labelled allogeneic Pan T-cells in a round-bottom 96 well-plate for 4 or 5 days, respectively. On day 4/5, cells were stained with CD3, CD4, CD8, and CD25 antibodies (dilutions see Table S6) and acquired by a BD FACSVerse or BD FACSLyric followed by analysis in FlowJo v.10 (Tree Star) to determine the frequency of proliferating CD4 and CD8 T-cells.

NY-ESO1 specific T cell activation assays

$CD1c^{+}CD14^{-}$ and $CD1c^{+}CD14^{+}$ cells were isolated and sorted as described previously from HLA-A*02:01^{+} (CD8 assay) or HLA-DRB1*04:01^{+} (CD4 assay) donors. A total of 10 000 cells were seeded in a round-bottom 96 well-plate, rested for 2–3 h, followed by treatment with MACS GMP PepTivator NY-ESO1 (Miltenyi, 170-076-137, consisting mainly of 15-mer peptides), $NY\text{-}ESO1_{p157\text{-}165}$ peptide (SLLMWITQC) for the CD8 assay, $NY\text{-}ESO1_{p117\text{-}143}$ peptide (PVPGVLLKEFTVSGNILTIRLTAADHR) for the CD4 assay, or $OVA_{257\text{-}164}$ (SIINFEKL) as irrelevant, negative control peptide. Meanwhile, 15 x 10^{6} autologous HLA-A*02:01 CD8 T-cells or HLA-DRB1*04:01^{+} CD4 T-cells, isolated as described above, were resuspended in 250 μL of RT phenol-free serum-free X-VIVO 15 (Lonza). 10 μg RNA encoding the α and β chains of the TCR recognizing the SLLMWITQC epitope presented on HLA-A*02:01 or, for CD4 T-cells, 10 μg RNA encoding the α and β chains of the TCR recognizing the PVPGVLLKEFTVSGNILTIRLTAADHR epitope presented on HLA-DRB1*04:01 was added to the cell suspension. Cells were transferred to electroporation cuvettes (1652088, BioRad), transfected using a Gene Pulser Xcell electroporation system (1652661, BioRad) with square wave, 500 V, 3 ms, 1 pulse, 4 mm, transferred to a 15 mL tube containing pre-warmed X-VIVO +2% HS, and left to recover (37°C, minimum of 2 h). T-cells were labeled with 2.5 μM CellTrace Violet dye (ThermoFisher) for 10 min at 37°C, followed by incubation in FCS for 30 min at 37°C. Cells were washed with PBS and counted.

After overnight incubation of $CD1c^{+}CD14^{-}$ and $CD1c^{+}CD14^{+}$ cells with the peptides, cells were washed and 50 000 TCR-transfected, CTV-labelled, CD8 or CD4 T-cells were added in a total volume of 200 μL. CD8 co-cultures were performed with 4 μg/mL R848 and 2 μg/mL poly I:C. After 24 h culturing supernatants were collected for subsequent cytokine quantification (see below). Cells were harvested after four days and stained for extracellular marker expression and analyzed using BD FACSLyric and FlowJo v.10 (Tree Start) software.

Cytokine quantification

6000 $CD1c^{+}CD14^{-}$ and $CD1c^{+}CD14^{+}$ cells, obtained by sorting as described previously, were cultured in 100 μL medium with and without 20 μg/mL poly I:C (Invivogen) and 4 μg/mL R848 (Invivogen) for 21 h. Culturing supernatants were collected and stored at −20°C until analysis by the LEGENDplex Human Inflammation panel 1 (Biolegend). Cytokine production by CD4 and CD8 NY-ESO1 specific T-cells was quantified using the LEGENDplex Human CD8/NK Panel (Biolegend), in 24 h culturing supernatant of co-cultured described above. LEGENDplex assays were performed according to manufacturer's instructions, acquired on MACSQuant Analyzer and data was analyzed using LEGENDplex Data Analysis Software.

PBMC cultures

PBMCs, isolated as described previously, were left untreated, depleted for $CD1c^{+}$ cells using the CD1c(BDCA1) DC isolation kit (Miltenyi, 130-119-475), or depleted for $CD1c^{+}$ and $CD34^{+}$ cells (using CD34 MicroBead Kit, Miltenyi 130-046-702). Cells were stained for extracellular marker expression and analyzed for the different CD1c-subset frequencies prior to culture and after a two-day culture in X-VIVO 15 with 2% HS with or without 50% BLM-CM.

OLINK analysis

Samples from 24h serum-free CM from BLM, Mel-624, A375, FM3, MeWo, Mel603, and A549 cells as well as serum from HDs and melanoma patients were sent to OLINK for simultaneous analysis of 96 protein biomarkers using the Olink Target 96 Immuno-Oncology panel (Table S5). The quality control was handled by Olink specialists. For serum samples, additional QC was performed by removing seven poorly detected proteins (<75% of samples) from further analyses. The limma package was used to detect differentially present proteins in cell line CM that did versus did not convert cDC2s to $CD14^{+}$-cDC2s. In this instance, no correction for multiple testing was performed according to the exploratory nature and small sample size. The volcano plot of cell line CM was created using the EnhancedVolcano package. Proteins that were unadjusted $p < 0.05$ and/or $\log_2 FC > 1$ were used to plot heatmaps (pheatmap) of scaled NPX values from cell line CM and serum analyses.

RNA microarray

Total RNA was extracted from FACS isolated $CD1c^{+}CD14^{-}$-cells, $CD1c^{+}CD14^{+}$-cells, and $CD14^{+}$-monocytes from blood of four HDs, using the RNeasy Plus Micro Kit (Qiagen). Samples were sent to Eurofins Genomics for RNA microarray analysis using the GeneChip 3′ IVT Pico Kit (Affymetrix) including initial RNA quality control measurements. Microarray data was normalized with RMA algorithm and further processed with R software. Differentially expressed genes (DEGs) were detected using the limma package (adjusted p value <0.05 (Benjamini Hochberg correction) with an absolute $\log_2$ FC cut-off of 2). Heatmap visualizing DEGs between the populations was created using heatmap.3 with hierarchical clustering according to Pearson correlation distance measure.

Drug treatment

Stattic (BioTechne, 2789) and Tofacitinib (Selleckchem, S2789) were dissolved in DMSO. To inhibit IL-6 signaling, cells were pre-incubated with 1 μM Stattic or Tofacitinib for 1 h at 37°C and 5% CO_2. After pre-incubation, 25 ng/mL human IL-6 (Miltenyi), 25 ng/mL human M-CSF (PeproTech), or BLM-CM (final concentration 50%) was added to the cells where indicated. After a 48 h culture period, cells were harvested and analyzed by flow cytometry. Sunitinib (Sutent, Pfizer) was suspended in DMSO to obtain a 1.43 mM stock solution. To inhibit IL6R and CSF1R signaling, cells were pre-incubated with 10 μg/mL tocilizumab (RoActemra, Roche) and/or 100 nM sunitinib, or 10 μg/mL Ultra-LEAF Purified anti-human M-CSF antibody (Clone A16067H, Biolegend) for 20 min at 37°C and 5% CO_2. Control samples were incubated with 10 μg/mL mouse IgG2b Isotype control or 10 μg/mL Ultra-LEAF Purified Human IgG1 Isotype control (Clone QA16A12, Biolegend) and/or DMSO as vehicle control. After pre-incubation, 1 ng/mL human IL-6 (Miltenyi), 10 ng/mL human M-CSF (PeproTech), or 50% BLM-CM was added to the cells where indicated. After a 48 h culture period, cells were harvested and divided for phenotype analysis by flow cytometry or re-seeded for allogeneic T cell proliferation assays.

QUANTIFICATION AND STATISTICAL ANALYSIS

Statistical analysis was performed in GraphPad Prism software (V8), unless otherwise indicated. Results are presented as mean ± SD in scatterplots (with bars), unless otherwise indicated in figure legends. For multiple group comparisons, one-way or two-way analysis of variance (ANOVA) or mixed-effects analysis was performed, followed by correction for multiple testing as indicated in each figure legend. Significance between two groups was tested with paired or unpaired Student's *t* tests. Statistical significance was annotated as: *p < 0.05, **p < 0.01, ***p < 0.001, ****p < 0.0001.

The following R packages were used in this study: the Tidyverse core packages,[47] openxlsx,[48] pheatmap,[49] EnhancedVolcano,[50] limma,[51] heatmap.3.[52]

ADDITIONAL RESOURCES

Included NSCLC patients are participants of the DONAN trial (NCT03853187): https://clinicaltrials.gov/ct2/show/NCT03853187.

Melanoma patients that received dendritic cells vaccinations were part of the Natural Dendritic Cell Vaccines in Metastatic Melanoma Patients trial (NCT01690377): https://clinicaltrials.gov/ct2/show/NCT01690377.

Cell Reports Medicine, Volume *5*

Supplemental information

Inhibition of CSF-1R and IL-6R prevents conversion of cDC2s into immune incompetent tumor-induced DC3s boosting DC-driven therapy potential

Anouk M.D. Becker, Annika H. Decker, Georgina Flórez-Grau, Ghaith Bakdash, Rutger J. Röring, Suzan Stelloo, Michiel Vermeulen, Berber Piet, Erik H.J.G. Aarntzen, Martijn Verdoes, and I. Jolanda M. de Vries

Supplemental information

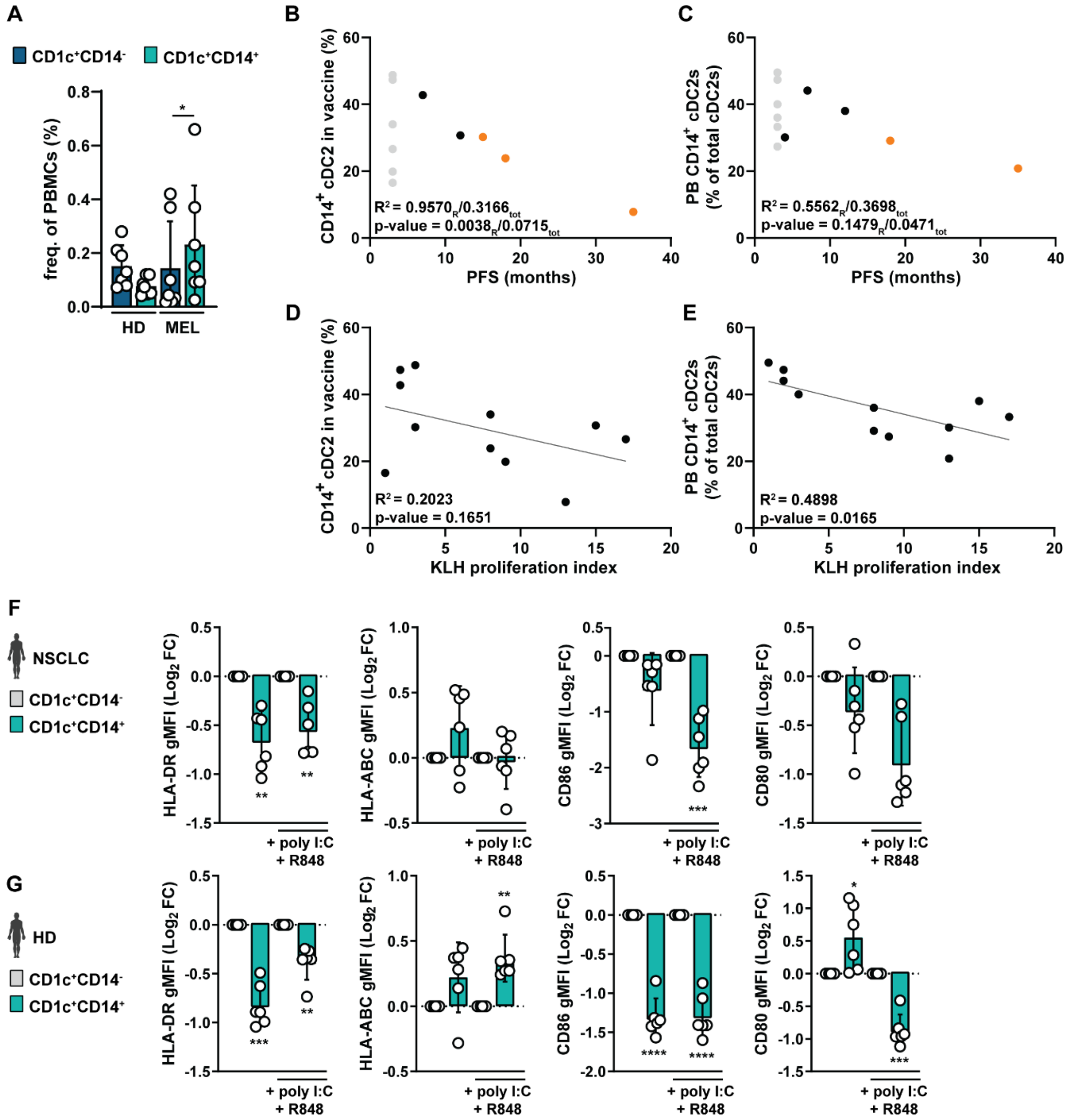

Figure S1. CD1c⁺CD14⁺ DCs affect DC vaccination efficacy and KLH-specific T cell proliferation in melanoma patients, and have lower co-stimulatory markers compared to CD1c⁺CD14⁻ cells in lung cancer patients and HDs. Related to Figure 1.

A Frequencies of $CD1c^+CD14^-$ and $CD1c^+CD14^+$ within PBMCs of healthy donors (HD) and melanoma patients (MEL). Each symbol shows a biological replicate with n=7 per group (mean±SD), two-way ANOVA with Sidak's multiple comparison test. **B** The fraction of $CD1c^+CD14^+$ in $CD1c^+$ DC-based vaccinations measured by flow cytometry for melanoma patients undergoing $CD1c^+$ DC-based vaccinations, correlated with the months of progression-free survival (PFS). **C** Correlation of PFS with the fraction of $CD1c^+CD14^+$ cells of total $CD1c^+$ (cDC2s) cells in peripheral blood (PB) of the same melanoma patients. Orange depicts patients with functional tumor antigen-specific T cells detected in peripheral blood or skin-infiltrating lymphocytes, grey indicates non-responder patients (all showing progressive disease). Correlation was statistically tested with Pearson's correlation coefficient and two-tailed P value, reported for responding patients($_R$)/all patients ($_{tot}$). Pearson's correlation coefficient of the anti-keyhole limpet hemocyanin (KLH) specific T cell proliferation from melanoma patients with **D)** the percentage of $CD1c^+CD14^+$ in $CD1c^+$ DC-based vaccinations and in **E)** the correlation with the fraction of $CD1c^+CD14^+$ cells of total $CD1c^+$ (cDC2s) cells in peripheral blood (PB) of melanoma patients. **B-D**

n=11 biological replicates (patients). PBMCs from **F)** NSCLC patients and **G)** healthy donors were depleted for CD3, CD19, CD56, using MACS beads followed by positive selection of CD1c$^+$ cells using the CD1c (BDCA1) DC isolation kit (Miltenyi). CD1c$^+$ cells were FACS sorted into CD1c$^+$CD14$^-$ and CD1c$^+$CD14$^+$ cells, cultured overnight with or without 20 µg/mL poly I:C and 4 µg/mL R848 and subsequently analyzed by flowcytometry for the surface expression of HLA-DR, HLA-ABC, CD86, CD80. Graphs show Log$_2$ FC of CD1c$^+$CD14$^+$ compared to CD1c$^+$CD14$^-$ cells, for immature and mature cDC2s. Each symbol represents an individual donor (mean±SD), with n=6 biological replicates for NSCLC and HD. Asterisks depict significance compared to CD1c$^+$CD14$^-$ cells in the same condition (two-tailed paired T-test), *p<0.05, **p<0.01, ***p<0.001, ****p<0.0001.

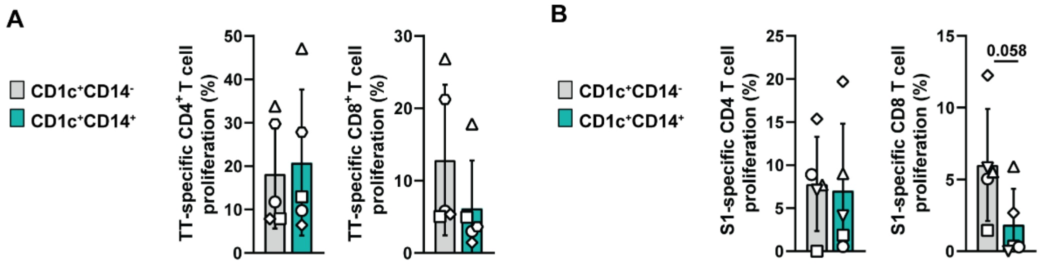

Figure S2. Antigen processing and presentation of cDC2 subsets in antigen-specific recall responses. Related to Figure 2.

CD1c$^+$CD14$^-$ and CD1c$^+$CD14$^+$ cells were isolated by FACS from healthy donors, rested overnight, and subsequently cultured with or without 1 µg/ml Tetanus Toxoid (TT, Sigma Aldrich) **(A)** or SARS-CoV-2 S Protein S1 (S1, Biolegend, 792904) **(B)** for 24 h. After 24 h, DCs were washed twice and co-cultured with CFSE-labelled autologous human pan T-cells (DC:T-cell ratio of 1:25) for 6 days after which T-cell proliferation was analyzed by flow cytometry. Antigen-specific T-cell proliferation was calculated by subtracting the percentage of proliferating T-cells measured in the conditions without antigen from the TT/S1 condition. Only donors showing an antigen-specific recall response were included in the analysis, each donor is represented by a different symbol with n=5 biological replicates each representing the average of technical duplicates (mean±SD, paired T-test).

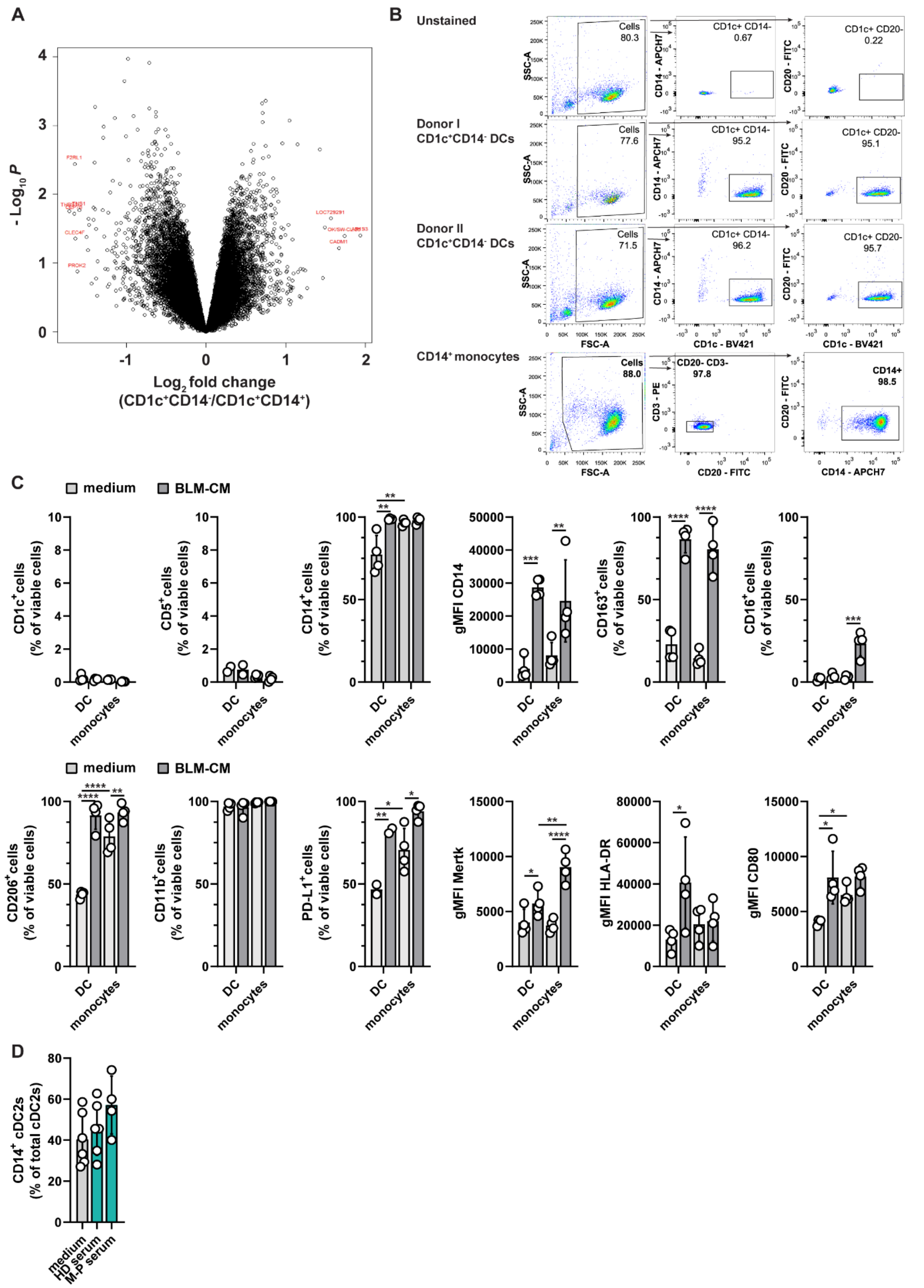

Figure S3. Tumor-induced CD1c⁺CD14⁺ cells derived from cDC2s phenotypically resemble macrophages after longer culture periods. Related to Figure 3.

A Volcano plot displaying expressed genes between CD1c⁺CD14⁻ and CD1c⁺CD14⁺ cells as analyzed by RNA Affymetrix Array Eurofins using n=4 biological replicates. Genes with an absolute FC > 1.5 are labeled, y-axis

depict non-adjusted P value, no genes were differentially expressed after correction for multiple testing. **B** Representative dot plots of purity analysis of $CD1c^{+}CD14^{-}$ cells and $CD14^{+}$ monocytes freshly isolated from human buffy coats, all stained with primary directly labelled antibodies: CD1c-BV421, CD20-FITC, CD3-PE and CD14-APC-H7 and assessed by flow cytometry. After MACS depletion of $CD19^{+}$ and $CD14^{+}$ cells, $CD1c^{+}$ cells were isolation by positive selection using the CD1c (BDCA1) DC isolation kit (Miltenyi). $CD14^{+}$ monocytes were directly isolated from PBMCs by positive selection using MACS CD14 beads (Miltenyi). **C** $CD14^{+}$ monocytes and $CD1c^{+}CD14^{-}$- DCs were isolated from HDs, cultured for seven days in X-VIVO (2% HS) with or without 50% BLM-conditioned medium (BLM-CM), and analyzed by flow cytometry (See also Table S4). Each symbol represents an individual donor (mean±SD, 2-way ANOVA with Sidak's multiple comparisons test for DCs versus monocytes and medium versus BLM-CM), with n=4 biological replicates. **D,** Frequencies of $CD1c^{+}CD14^{+}$ cells after a three-day culture of $CD14^{-}$ cDC2s from healthy donors (HDs) with HD serum or melanoma patients (M-P) serum. Each symbol represents an individual serum donor or DC donor for the medium condition, with n=6 biological replicates for medium, n=6 for HD serum and n=4 for serum from M-P, each tested on a minimum of n=2 DC-donors.

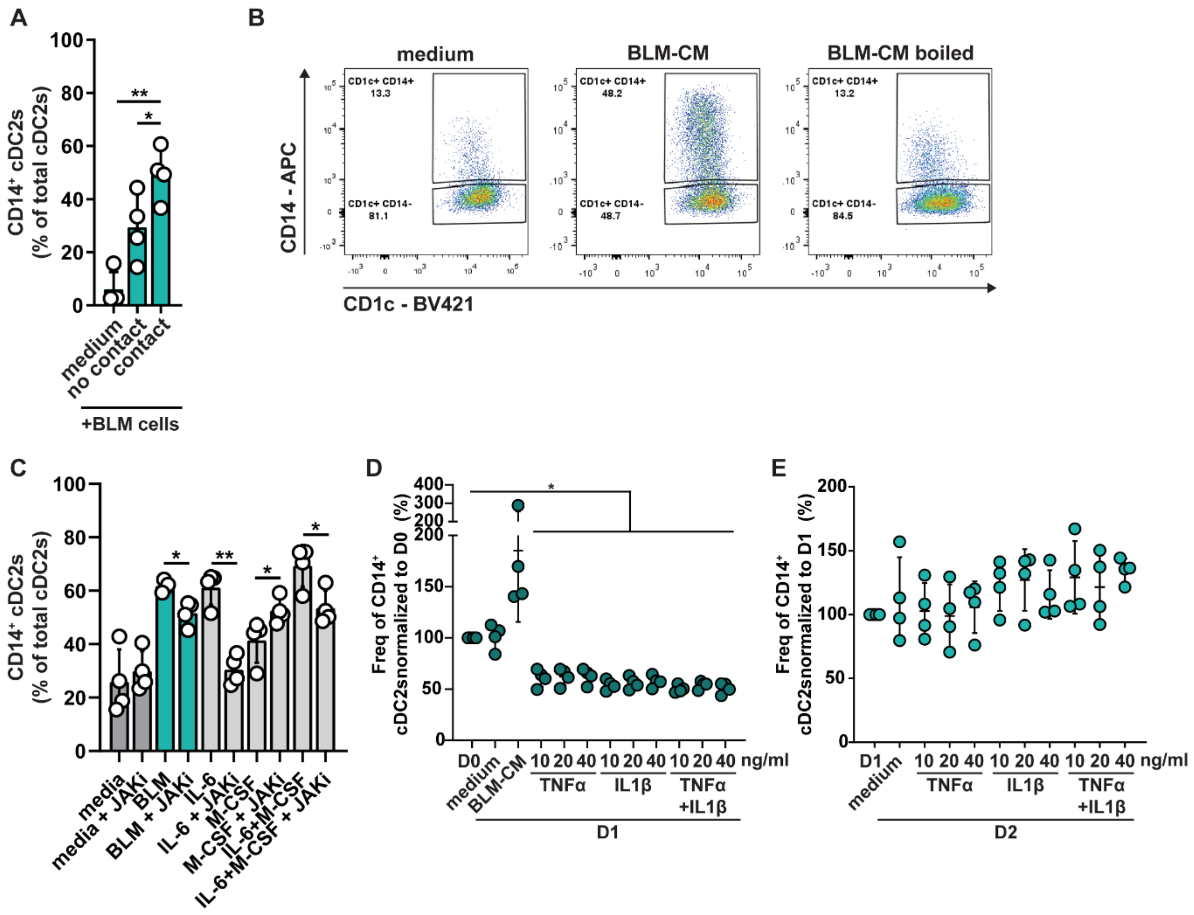

Figure S4. CD14⁺ cDC2s are irreversibly induced by soluble tumor-associated proteins. Related to Figure 4 and 5.

A Frequencies of CD14⁺ cDC2s after culturing CD14⁻ cDC2s from healthy donors in a transwell plate with or without direct cell-cell contact with BLM cells, n=4 biological replicates depicted by each symbol. **B** CD14⁻ cDC2s from healthy donors were cultured in the presence of 40% BLM-conditioned medium (CM) either untreated or heated at 95 °C for 10 minutes. After three days, cultured cells were analyzed by flow cytometry for CD14 and CD1c expression. Shown are representative dot-plots of n=3 biological replicates. **C** Induction of CD14⁺ cDC2s in the absence or presence of 1 µM Janus kinase inhibitor (JAKi) Tofacitinib after a two-day culture period with 50% BLM-CM, 25 ng/mL IL-6 and/or 25 ng/mL M-CSF. Each symbol represents a biological replicate (n=4), mean±SD, one-way RM ANOVA with Dunnett's multiple comparisons test between untreated and JAKi treated for each condition. **D+E** Phenotypic plasticity of CD14⁺cDC2s analyzed for CD14⁺ cDC2s from healthy donors **(D)** and for BLM-CM induced CD14⁺ cDC2s **(E)**. Graphs show frequencies of CD14⁺cDC2s normalized to D0 **(D)** and normalized to D1 after inducing CD14⁺ cDC2s with BLM-CM for 24 hours **(E),** both with the indicated concentration range of TNFα and/or IL1β. Each symbol represents an individual donor, with n=4 biological replicates for each experiment (mean±SD). Asterisks depict significance compared to D0 **(D)** or D1 **(E)** (RM one-way ANOVA, Dunnett's multiple comparisons test). *p<0.05, **p<0.01, ***p<0.001

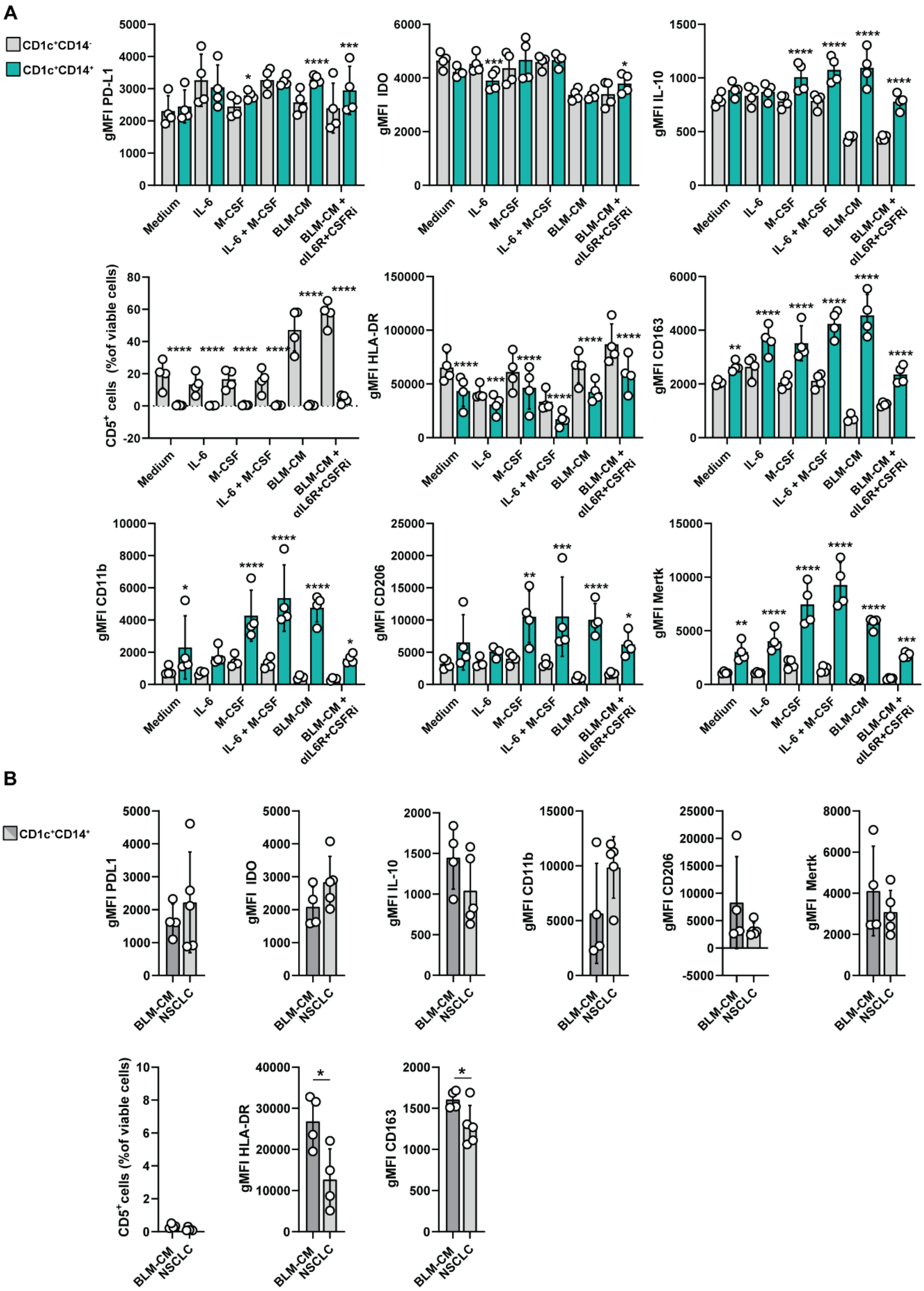

Figure S5. *In vitro* tumor induced CD14⁺ cDC2s phenotypically resemble CD14⁺ cDC2s from lung cancer patients. Related to Figure 6.

A $CD14^-$ cDC2s isolated from HDs were cultured for three days in depicted conditions and expression of phenotypic hallmarks was assessed by flow cytometry and compared between induced $CD14^+$ cDC2s and the remaining $CD14^-$ cDC2s in culture. Each symbol represents an individual donor (mean ± SD) with n=4 biological replicates, asterisks depict significant results compared to $CD14^-$ cDC2s (2-way RM ANOVA, Sidak's multiple

comparisons test). **B** Phenotype comparison between BLM-conditioned medium (CM) induced $CD14^+$ cDC2s from HDs and $CD14^+$ cDC2s isolated from NSCLC patients, both cultured for three days (unpaired t-test, HD n=4, NSCLC n=5 biological replicates, mean ± SD). *p<0.05, **p<0.01, ***p<0.001, ****p<0.0001

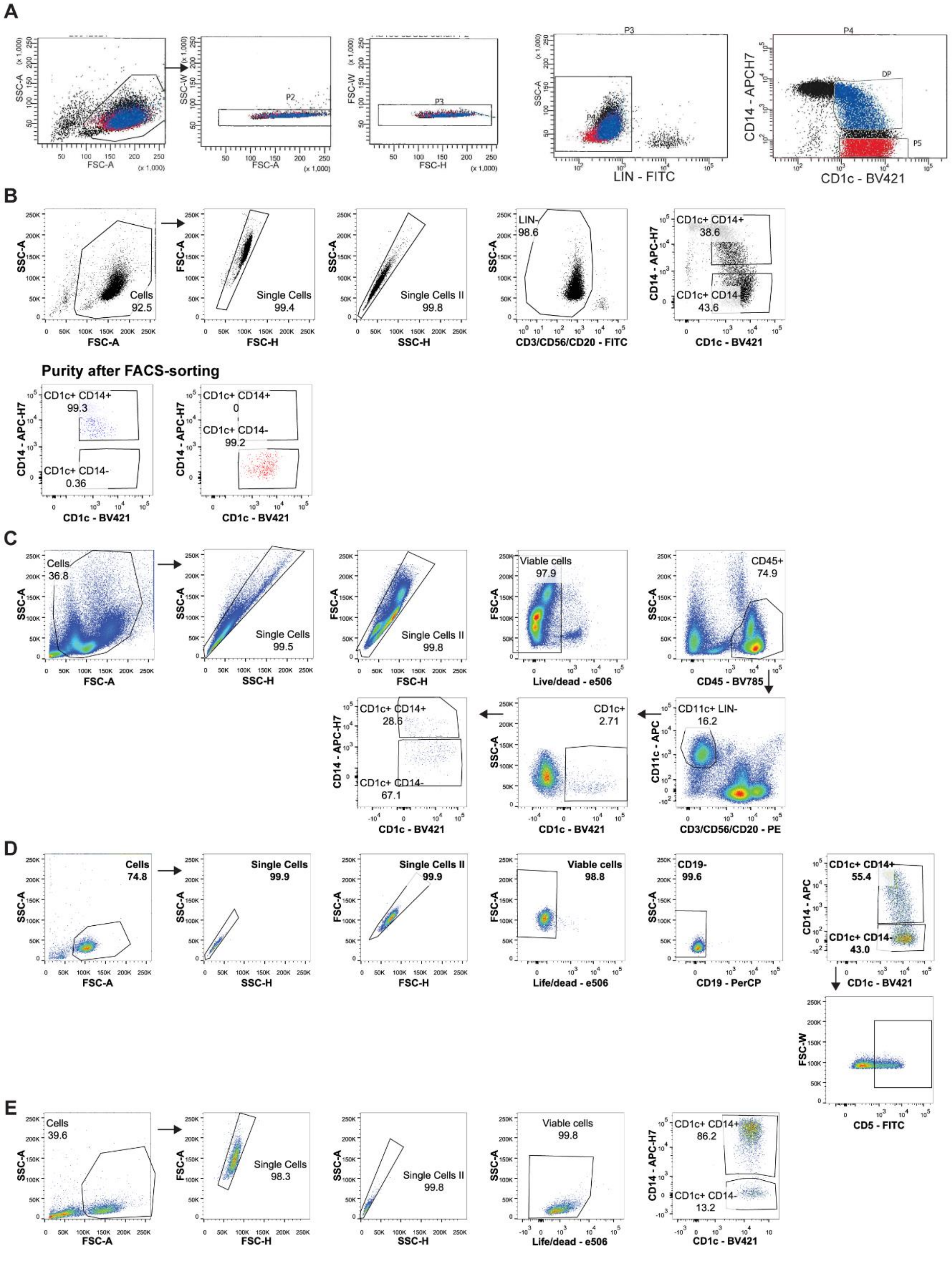

Figure S6. Gating strategies. Related to STAR Methods, Figure 1-6, Table S4.

Manual gating strategy to define and sort CD1c^{+}CD14^{-} and CD1c^{+}CD14^{+} cells after lineage depletion performed on BD FACS Aria **(A)** or Melody **(B)**, with two representative purity plots. Sorted cells were used for bulk RNA microarray analysis, T-cell assays, and cytokine profiling. **C** Gating strategy to define CD1c^{+}CD14^{-} and CD1c^{+}CD14^{+} cells within peripheral blood mononuclear cells. Representative examples of the gating strategy for flow cytometry analysis of isolated CD1c^{+} cells is shown in **D)** for samples immediately stained after isolation (some panels include CD14-APC-H7 and CD20-FITC instead of CD14-APC and CD19-PerCP, respectively, see table S4) and in **E)** for CD1c^{+} cells after a culture period. See also table S4.

Table S1. Patient information melanoma patients DC-vaccination trial. Related to STAR Methods, Fig. S1A-E, 1A.

ID	disease (stage)	gender	year of birth
**Melanoma patients DC-vaccination trial (Fig. S1B-E)**			
VI-B-01	melanoma (M1a)	M	1958
VI-B-02	melanoma (M1c)	M	1945
VI-B-03	melanoma (M1a)	F	1968
VI-B-04	melanoma (M1c)	M	1985
VI-B-05	melanoma (M1c)	F	1974
VI-B-06	melanoma (M1c)	M	1964
VI-B-08	melanoma (M1c)	F	1962
VI-B-09	melanoma (M1a)	M	1947
VI-B-10	melanoma (M1c)	F	1950
VI-B-11	melanoma (M1c)	M	1968
VI-B-12	melanoma (M1b)	M	1970
VI-B-13	melanoma (M1b)	M	1970
VI-B-14	melanoma (IIIcb)	M	1943
**Melanoma patients PBMC assessment (Fig. 1A, Fig. S1A)**			
IPI02	melanoma	F	1942
IPI08	melanoma	F	1961
IPI09	melanoma	F	1946
IPI11	melanoma	M	1958
IPI12	melanoma	M	1952
IPI13	melanoma	F	1963
IPI14	melanoma	F	1945

Table S2. Patient information non-small cell lung cancer (NSCLC) patients. Related to STAR Methods, Fig. 1, 2A-B, 3A-B

ID	disease	gender	year of birth
DON240	NSCLC	F	1968
DON395	NSCLC	F	1969
DON014	NSCLC	F	1948
DON092	NSCLC	M	1947
DON609	NSCLC	F	1969
DON986	NSCLC	M	1964
DON104	NSCLC	F	1962
DON469	NSCLC	F	1949
DON344	NSCLC	M	1952
DON622	NSCLC	F	1963
DON430	NSCLC	M	1949
DON570	NSCLC	F	1957
DON166	NSCLC	F	1948
DON159	NSCLC	M	1948
DON109	NSCLC	M	1944
DON590	NSCLC	M	1957

Table S3. Absolute cytokine amounts produced by CD1c⁺CD14⁻ and CD1c⁺CD14⁺ cells as measured by LEGENDPlex. Related to figure 2A-B. Shown are average results of HD (n=5) and non-small cell lung cancer (NSCLC) patients (n=6). IFNα and IFNγ levels were below detection limits. DC2: $CD1c^{+}CD14^{-}$, DC3: $CD1c^{+}CD14^{+}$

	IL-6	IL-1β	TNFa	MCP-1	IL-8	IL-10	IL12-p70	IL-17	IL-18	IL-23	IL-33
**HD - DC2**	21.33	3.28	3.09	3.45	5710	2.25	1.30	0.16	10.18	6.16	5.90
**HD - DC3**	2395	380.3	235.10	83.46	12000	104.4	36.64	0.25	27.83	144	7.84
**NSCLC - DC2**	54.93	1.68	4.43	5.15	3287	1.92	1.03	0.13	6.55	5.76	5.00
**NSCLC - DC3**	468.5	62.55	35.73	144.4	4900	14.43	1.38	0.16	10.93	10.5	6.07
**HD mature DC2**	7687	1117	5428	16.24	12000	478.1	758.6	0.39	38.83	1658	17.76
**HD mature DC3**	7766	2880	6938	49.95	12000	392.2	194.1	0.36	51.50	3068	24.94
**NSCLC mature DC2**	4020	541.8	2966	237.10	12000	394.2	467.9	0.21	26.10	837.4	15.59
**NSCLC mature DC3**	5045	1942	4217	1189	12000	379.6	72.3	0.29	44.56	1312	17.38

[a]blue and red indicate lower and upper detection limit, respectively

Table S4. FACS panels used in the study. See also Figure S6.

	Laser		Violet (405nm)				Blue (488nm)				Red (633nm)	
	Band pass filter		448/45	528/45	606/36	755 LP	527/32	586/42	700/54	783/56	660/10	783/56
**Panel**	**Data in**	**Cytometer**										
**cDC2 frequencies in PBMCs**	fig. 1A, 2A,B	Lyric	CD1c BV421	L/D e506		CD45 BV785		CD3/CD56/CD20 PE			CD11c APC	CD14 APC-H7
**cDC2 phenotype HD I**	fig. 1C,D,E, fig. S5	Verse	CD1c BV421	L/D e506			CD20 FITC	CD206 PE	HLA-DR PerCP	MERTK PE-Cy7	CD14 APC	CD11b APC-Cy7
**cDC2 phenotype HD II**	fig. 1C,D,E, fig. S5	Verse	CD1c BV421	L/D e506			CD5 FITC	CD163 PE	CD19 PerCP	PD-L1 PE-Cy7		CD14 APC-H7
**cDC2 phenotype HD III**	fig. 1C,D,E, fig. S5	Verse	CD1c BV421	L/D e506			***IDO AF488***		CD19 PerCP	***IL-10 PE-Cy7***	CD14 APC	
**cDC2 cultured**	fig. 3E,F fig. 4A,C,F fig. 5, fig. 6B,C,E, fig. S3D, S4	Verse	CD1c BV421	L/D e506					HLA-DR PerCP		CD14 APC[a]	CD14 APC-H7[a]
**Purity after isolation**	fig. S3B	Verse	CD1c BV421	L/D e506			CD20 FITC	CD3 PE				CD14 APC-H7
**Sort panel for Aria**	fig. 1E/fig. 3C	Aria	CD1c BV421				CD3/CD56/CD20 FITC					CD14 APC-H7
**Sort panel for Melody**	fig. 1E, fig. 2	Melody					CD3/CD56/CD20 FITC	CD1c PE				CD14 APC-H7
**T cell proliferation panel**	fig. 1E,6D, fig. S2	Lyric	CD4 BV421	CD3 BV510			CFSE			CD25 PE-Cy7	CD8 APC	L/D e780
**Maturation markers HD**	fig. S1G	Verse	CD1c BV421	HLA-DR BV510			HLA-ABC FITC		CD80 PerCP-eFluor710		CD86 APC	CD14 APC-H7
**Maturation markers NSCLC**	fig. S1F	Verse	CD1c BV421	L/D e506			HLA-ABC FITC	CD86 PE	HLA-DR PerCP	CD80 PE-Cy7		CD14 APC-H7
**CD8 NY-ESO1 assay**	fig. 2C,D	Lyric	CTV		CD8 BV605			PD-1 PE		CD25 PE-Cy7		L/D e780
**CD4 NY-ESO1 assay**	fig. 2F,G	Lyric	CTV			CD4 BV786	CD127 FITC			CD25 PE-Cy7		L/D e780
**D7 monocytes DCs I**	fig. S3C	Lyric	CD1c BV421	L/D e506	CD163 BV605		CD206 FITC	CD11b PE	HLA-DR PerCP	PD-L1 PE-Cy7		CD14 APC-H7

**D7 monocytes DCs II**	fig. S3C	Lyric	CD1c<br>BV421	L/D<br>e506	CD163<br>BV605		CD5<br>FITC	CD80<br>PE	CD16<br>PerCP-Cy5.5	MERTK<br>PE-Cy7		CD14<br>APC-H7
**PBMC purity +cultured**	fig. 3G,H	Lyric	CD1c<br>BV421	L/D<br>e506	CD163<br>BV605	CD11c<br>BV785	CD34<br>FITC	CD3/CD56/CD20<br>PE				CD14<br>APC-H7

[a] CD14 APC or CD14 APC-H7 was used
[b] Intracellular staining are italicized and in green

Table S6. Dilutions for the antibodies used in the study and reported in the Key resource table. Related to Figures 1-6.

Marker	Fluorochrome	Clone	Dilution	RRID identifier
CD45	BV785	HI30	1 in 25	(BioLegend Cat# 304048, RRID:AB_2563129)
CD11b	APC cy7	ICRF44	1 in 40	(BioLegend Cat# 301342, RRID:AB_2563395)
CD11c	APC	B-ly6	1 in 20	(BD Biosciences Cat# 559877, RRID:AB_398680)
CD14	APC	M5E2	1 in 40	(BioLegend Cat# 301808, RRID:AB_314190)
CD14	APC-H7	MφP9	1 in 30	(BD Biosciences Cat# 560180, RRID:AB_1645464)
CD163	PE	GHI/61	1 in 25	(BD Biosciences Cat# 556018, RRID:AB_396296)
CD19	PerCP	4G7	1 in 30	(BD Biosciences Cat# 345778, RRID:AB_2868806)
CD1c	BV421	L161	1 in 30	(BioLegend Cat# 331526, RRID:AB_10962909)
CD1c	PE	AD5-8E7	1 in 50	(Miltenyi Biotec Cat# 130-113-302, RRID:AB_2726081)
CD20	FITC	L27	1 in 10	(BD Biosciences Cat# 345792, RRID:AB_2868818)
CD206	PE	19.2(RUO)	1 in 10	(BD Biosciences Cat# 555954, RRID:AB_396250)
CD5	FITC	L17f12	1 in 100	(Thermo Fisher Scientific Cat# 11-0058-42, RRID:AB_1944383)
HLA-DR	PerCP	L243	1 in 30	(BioLegend Cat# 307628, RRID:AB_893566)
HLA-DR	BV510	L243	1 in 20	(BioLegend Cat# 307646, RRID:AB_2561948)
HLA-ABC	FITC	REA230	1 in 25	(Miltenyi Biotec Cat# 130-101-446, RRID:AB_2652080)
CD80	PE-Cy7	L307.4	1 in 20	(BD Biosciences Cat# 561135, RRID:AB_10561688)
CD80	PerCP-eFluor710	2D10.4	1 in 30	(Thermo Fisher Scientific Cat# 46-0809-42, RRID:AB_10548359)
CD86	PE	FUN-1	1 in 20	(BD Biosciences Cat# 555658, RRID:AB_396013)
CD86	APC	FUN-1	1 in 20	(BD Biosciences Cat# 555660, RRID:AB_398608)
IDO1	AF488	#700838	1 in 20	(R and D Systems Cat# IC6030G, RRID:AB_10997134)
IL-10	PE-Cy7	JES3-9D7	1 in 20	(BioLegend Cat# 501420, RRID:AB_2125385)
Mertk	PE-Cy7	590H11G1E3	1 in 30	(BioLegend Cat# 367609, RRID:AB_2687286)
PD-L1	PE-Cy7	MIH1	1 in 20	(BD Biosciences Cat# 558017, RRID:AB_396986)
CD3	FITC	HIT3a	1 in 10	(BD Biosciences Cat# 555339, RRID:AB_395745)
CD56	FITC	NCAM16.2	1 in 10	(BD Biosciences Cat# 345811, RRID:AB_2868832)
CD4	BV421	RPA-T4	1 in 50	(BD Biosciences Cat# 562424, RRID:AB_11154417)
CD8	APC	RPA-T8	1 in 50	(BD Biosciences Cat# 555369, RRID:AB_398595)
CD8	BV605	G42-8	1 in 50	(BD Biosciences Cat# 743066, RRID:AB_2741260)
CD25	PE-Cy7	BC96	1 in 30	(BioLegend Cat# 302612, RRID:AB_314282)
CD3	BV510	SK7	1 in 50	(BioLegend Cat# 344828, RRID:AB_2563704)
CD3	PE	HIT3a	1 in 25	(BD Biosciences Cat# 555340, RRID:AB_395746)
CD56	PE	5.1H11	1 in 25	(BioLegend Cat# 981202, RRID:AB_2715758)
CD20	PE	2H7	1 in 20	(BioLegend Cat# 302306, RRID:AB_314254)
PD-1	PE	MIH4	1 in 25	(BD Biosciences Cat# 557946, RRID:AB_647199)
CD4	BV786	SK3	1 in 50	(BD Biosciences Cat# 563877, RRID:AB_2738462)
CD127	FITC	A019D5	1 in 20	(BioLegend Cat# 351312, RRID:AB_10897643)
CD163	BV605	GHI/61	1 in 20	(BioLegend Cat# 333616, RRID:AB_2616879)
CD206	FITC	19.2	1 in 10	(BD Biosciences Cat# 551135, RRID:AB_394065)
CD11b	PE	ICRF44	1 in 25	(BioLegend Cat# 301306, RRID:AB_314158)

CD80	PE	L307.4	1 in 10	(BD Biosciences Cat# 557227, RRID:AB_396606)
CD16	PerCPCy5.5	3G8	1 in 20	(BD Biosciences Cat# 560717, RRID:AB_1727434)
CD11c	BV785	3.9	1 in 20	(BioLegend Cat# 301644, RRID:AB_2565779)
CD34	FITC	561	1 in 20	(BioLegend Cat# 343604, RRID:AB_1732005)
